# Cardiac abnormalities in Long COVID 1-year post-SARS-CoV-2 infection

**DOI:** 10.1136/openhrt-2022-002241

**Published:** 2023-02-23

**Authors:** Adriana Roca-Fernandez, Malgorzata Wamil, Alison Telford, Valentina Carapella, Alessandra Borlotti, David Monteiro, Helena Thomaides-Brears, Matt Kelly, Andrea Dennis, Rajarshi Banerjee, Matthew Robson, Michael Brady, Gregory Y H Lip, Sacha Bull, Melissa Heightman, Ntobeko Ntusi, Amitava Banerjee

**Affiliations:** 1 Perspectum Ltd, Oxford, UK; 2 Department of Cardiology, Great Western Hospital Foundation NHS Trust, Swindon, UK; 3 Department of Cardiology, Oxford Radcliffe Hospitals NHS Trust, Oxford, UK; 4 Liverpool Centre for Cardiovascular Science, University of Liverpool, Liverpool, UK; 5 Department of Cardiology, Royal Berkshire Hospital NHS Foundation Trust, Reading, UK; 6 Department of Respiratory Medicine, University College London Hospitals NHS Trust, London, UK; 7 Medicine, University of Cape Town, Cape Town, South Africa; 8 Institute of Health Informatics, University College London, London, UK; 9 Department of Cardiology, University College London Hospitals NHS Trust, London, UK; 10 Department of Cardiology, Barts Health NHS Trust, London, UK

**Keywords:** COVID-19, Myocarditis, Magnetic Resonance Imaging, EPIDEMIOLOGY

## Abstract

**Background:**

Long COVID is associated with multiple symptoms and impairment in multiple organs. Cross-sectional studies have reported cardiac impairment to varying degrees by varying methodologies. Using cardiac MR (CMR), we investigated a 12-month trajectory of abnormalities in Long COVID.

**Objectives:**

To investigate cardiac abnormalities 1-year post-SARS-CoV-2 infection.

**Methods:**

534 individuals with Long COVID underwent CMR (T1/T2 mapping, cardiac mass, volumes, function and strain) and multiorgan MRI at 6 months (IQR 4.3–7.3) since first post-COVID-19 symptoms. 330 were rescanned at 12.6 (IQR 11.4–14.2) months if abnormal baseline findings were reported. Symptoms, questionnaires and blood samples were collected at both time points. CMR abnormalities were defined as ≥1 of low left or right ventricular ejection fraction (LVEF), high left or right ventricular end diastolic volume, low 3D left ventricular global longitudinal strain (GLS), or elevated native T1 in ≥3 cardiac segments. Significant change over time was reported by comparison with 92 healthy controls.

**Results:**

Technical success of multiorgan and CMR assessment in non-acute settings was 99.1% and 99.6% at baseline, and 98.3% and 98.8% at follow-up. Of individuals with Long COVID, 102/534 (19%) had CMR abnormalities at baseline; 71/102 had complete paired data at 12 months. Of those, 58% presented with ongoing CMR abnormalities at 12 months. High sensitivity cardiac troponin I and B-type natriuretic peptide were not predictive of CMR findings, symptoms or clinical outcomes. At baseline, low LVEF was associated with persistent CMR abnormality, abnormal GLS associated with low quality of life and abnormal T1 in at least three segments was associated with better clinical outcomes at 12 months.

**Conclusion:**

CMR abnormalities (left entricular or right ventricular dysfunction/dilatation and/or abnormal T1mapping), occurred in one in five individuals with Long COVID at 6 months, persisting in over half of those at 12 months. Cardiac-related blood biomarkers could not identify CMR abnormalities in Long COVID.

**Trial registration number:**

NCT04369807.

**WHAT IS ALREADY KNOWN ON THIS TOPIC**

- Acute COVID-19 can be associated with various cardiovascular complications, including myocarditis, ventricular disfunction or acute coronary syndrome, however, the evolution of cardiac impairment, especially in non-hospitalised patients has not been fully investigated.

**WHAT THIS STUDY ADDS**

- We specify the nature of cardiac abnormalities in Long COVID, linked to clinical characteristics at 1 year. Within a multiorgan context, we provide a holistic view of Long COVID assessment, developed in a community cohort of mainly non-hospitalised individuals with varying severity of symptoms.

**HOW THIS STUDY MIGHT AFFECT RESEARCH, PRACTICE OR POLICY**

- Comprehensive cardiac MRI assessment may guide clinical decision making and improve healthcare resource utilisation. Evidence of cardiac involvement could inform follow-up assessment and identification of Long COVID subtypes in research and practice, as well as interventional trials to evaluate cost-effective therapies.

## Introduction

Cardiovascular disease is linked to COVID-19 severity and mortality since the first reports from Wuhan in late 2019.^1–3^ However, associations between Long COVID symptoms and cardiac impairment are unclear, and the subtypes more likely to recover have not been identified.

In a large post-COVID-19 assessment service in the UK, almost half of individuals where cardiac MR (CMR) scans were performed had evidence of mild myocarditis^4^ and in a smaller study, symptom improvement at 6 months was neither correlated with improvement on CMR imaging nor lung parenchymal recovery.^5^ A systematic review of CMR findings post-COVID-19 identified myocarditis as the most prevalent diagnosis (14%),^6^ though not all classical features are evident on biopsy,^7 8^ and T1 abnormalities and oedema on T2 as the most common findings, and occasional late gadolinium enhancement (LGE).^8^ These findings may be present even in absence of elevated cardiac blood biomarkers (eg, troponin or NT-pro-BNP, natriuretic peptide pro B-type natriuretic peptide).^6 9 10^ Pericardial effusion and reduced LV and RV function have been occasionally reported, but pericarditis is rare. Nevertheless, to date there is no clear definition of cardiac change post-COVID-19 and cardiac abnormalities in Long COVID at baseline and over time are ill defined in the community setting.

Although echocardiography is often the first choice for assessment of cardiac function, CMR is the gold-standard assessment, ensuring a more accurate assessment of cardiac structure and function. We; therefore, conducted a prospective, longitudinal 1-year study using CMR alongside multiorgan MRI assessment, in the largest Long COVID community cohort available to date, to investigate: (1) The evolution of cardiac abnormalities over 1 year after SARS-CoV-2 infection in a multiorgan context; (2) the prevalence and severity of cardiac abnormalities in the non-hospitalised versus the hospitalised population and (3) the associations to patient outcomes that could be used to guide clinical pathway design and identification of at risk individuals.

## Methods

### Population and study design

**Figure 1:**
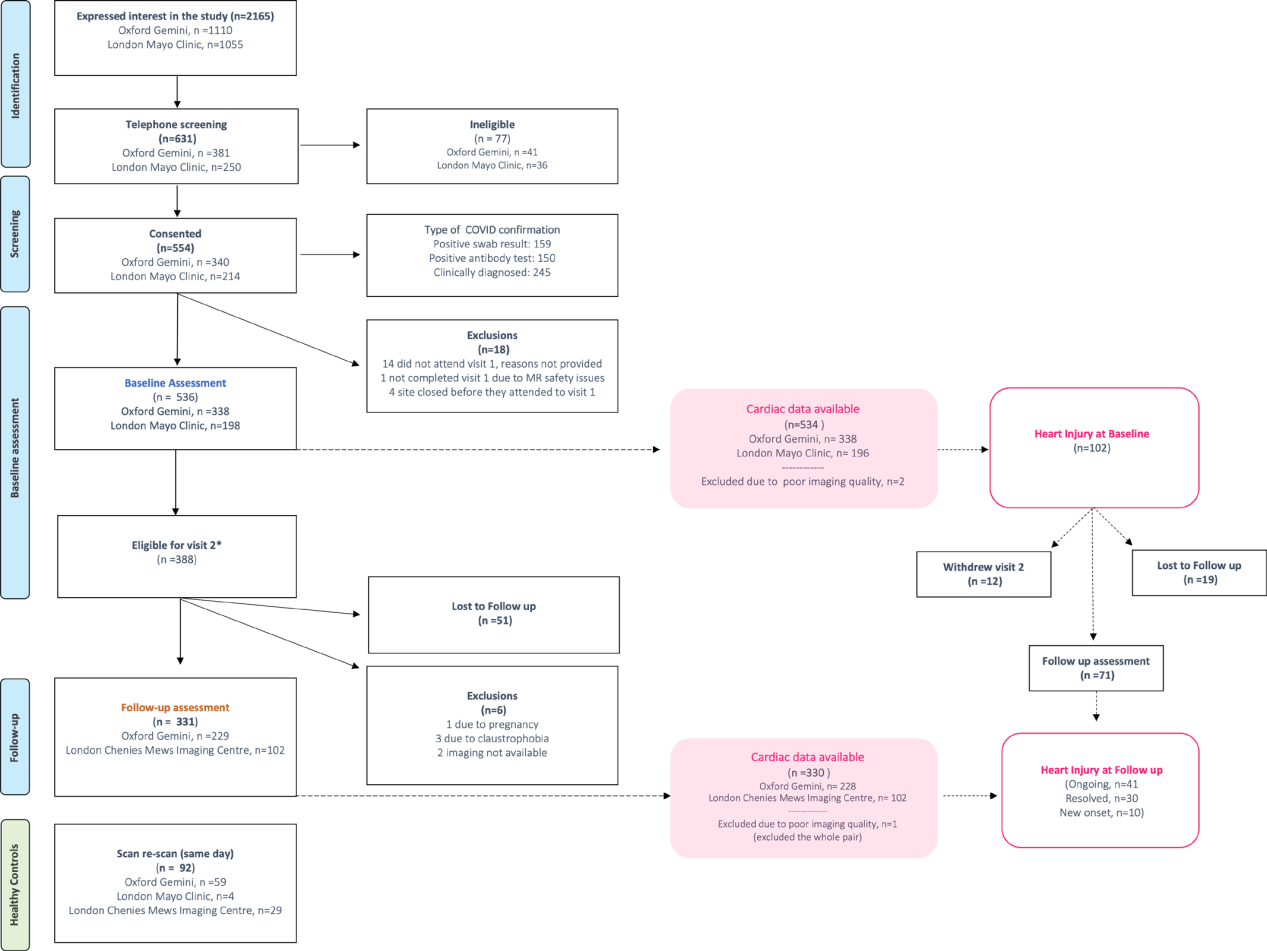
Study population for cardiac complications of long COVID. *Individuals were eligible for follow up when MRI abnormality or abnormal bloods in any organ were found at baseline.

The COVERSCAN study (NCT04369807) is a prospective study of organ function using quantitative MRI in individuals recovering from SARS-CoV-2 infection with persistent COVID-19 symptoms in a community setting. Individuals were recruited via advertisement, including in Long COVID support groups and hospital referral (online supplemental methods 1), and invited to undergo CoverScan (Perspectum, Oxford, UK), a multiparametric MRI assessment of lungs, heart, liver, pancreas, kidneys and spleen. All imaging assessments were performed at Perspectum (Oxford), Mayo Clinic (London) and Chenies Mews Imaging Centre (London), between April 2020 and October 2021 (figure 1). Healthy controls were recruited within the same period, based on self-reporting medical history, and scanned twice on the same date to derive reference ranges and assess repeatability. COVID-19 was classified by either laboratory-confirmed SARS-CoV-2 infection (159 tested SARS-CoV-2-positive by oropharyngeal/nasopharyngeal swab for reverse-transcriptase PCR; 150 individuals with positive antibodies) or strong clinical suspicion of SARS-CoV-2 infection with typical symptoms/signs confirmed by 2 clinicians (245 individuals). Exclusion criteria were symptoms of active respiratory viral infection (temperature >37.8°C or ≥3 episodes of coughing in 24 hours), hospital discharge in the last 7 days and contraindications to MRI, including implanted pacemakers, defibrillators, other metallic implanted devices and claustrophobia. Participants gave written informed consent. Those with organ abnormality at baseline MRI scan (in ≥1 of the following organs: lungs, heart, liver, pancreas, spleen, kidneys) or blood tests were invited back for 6-month follow-up, corresponding to 1-year postinfection. Incidental findings classified as benign and/or not requiring follow-up by an experienced radiologist were not invited for follow-up.

10.1136/openhrt-2022-002241.supp1 Supplementary data

### Symptoms, quality of life and function

Presence and severity of symptoms were assessed by self-report and validated questionnaires: EQ-5D-5L (EuroQoL-5 dimension-5 level; utility score and quality of life related to usual activities), and Dyspnoea-12 at baseline and follow-up, when Left Ventricular Dysfunction Questionnaire (LVD-36) was also conducted (online supplemental methods 2). For self-reported symptoms at baseline, participants were asked to report only new symptoms arising since the COVID infection; at follow-up, they were asked to report symptoms since baseline. Time off work due to Long COVID was recorded as total number of days at follow-up.

### Blood investigations

Two blood samples were taken at both timepoints, on the same day as the MRI scan: one immediately sent for analysis, the other fractionated and frozen for later analysis (online supplemental methods 3).

### Multiorgan imaging

Participants were scanned at Perspectum Gemini (Oxford: n=338; MAGNETOM Aera 1.5T scanner) and Mayo Clinic (London: n=198; MAGNETOM Vida 3T) (both scanners: Siemens Healthcare, Erlangen, Germany), at baseline and follow-up with multiorgan, multiparametric MRI assessment (total ~40 min duration). All imaging methods were deployed in standard clinical MRI scanners using slightly modified versions of previously published methods^11 12^ and using short (<14 s) breath-holds except for lung imaging (online supplemental methods 4 and 5).

After each visit, participants and if requested their primary care physicians also, received a clinical summary and a report informing on the MRI data, where quantitative metrics were referenced against the healthy control population, and one on the blood biomarker data.

### Reference ranges and repeatability coefficients

In parallel, 92 sex-matched and age-matched healthy individuals (online supplemental methods 6 tables S1, S2) were recruited and scanned twice on the same day, to derive a control group. Reference ranges using the healthy control population were calculated for each metric by computing 2.5% and 97.5% percentiles using bootstrapping (100 000 permutations), except pancreas proton-density fat fraction (PDFF), where the 95% percentile was for the upper limit, and liver cT1 and PDFF, where we used established thresholds.^13^ Reference ranges for organ length and volume required larger sample size for sex and height stratification, so we used a sample of 1836 individuals from UK Biobank without self-reported diabetes or hypertension. To evaluate measurement repeatability, two separate scans were performed in healthy controls (1.5T, n=59; 3T, n=33) on the same day. After first scan, the participant had a 10 min break out of the scanner before a second identical scan. Technical success was assessed by quality-assured measures for each variable, and overall, in report delivery for each patient (online supplemental table S1).

### Definition of cardiac and multiorgan abnormality

CMR abnormalities were defined by consensus among expert cardiologists with experience of Long COVID patients and following literature review of common cardiac findings post-COVID-19 as: ≥1 of the following outside reference range left or right ventricular ejection fraction (LVEF or RVEF) or left or right ventricular end diastolic volume, global longitudinal strain (GLS) (abnormal will be referred as low, in absolute values) or ≥3 quantitative T1 mapping segments. Two cardiologists independently reviewed all CMR findings ahead of statistical analysis in this work. Multiorgan impairment was defined as ≥2 measurements outside reference ranges in a further organ (excluding elevated liver or kidney volume)^11^ (further details in online supplemental methods 5 table S1).

### Statistical analysis

We used R software V.4.0.4 and p values <0.05 defined statistical significance. Normality was assessed using Shapiro test. To describe parametric and non-parametric variables, we used mean (SD) and median (IQR), respectively. For categorical variables, we reported frequencies (percentage). For groupwise comparisons of continuous parametric and non-parametric, and categorical variables, t-test, Wilcoxon rank sum and Fisher’s exact tests, respectively, were used, without correction for multiple testing as analyses were exploratory. Baseline and follow-up metrics were assessed using reference ranges calculated in healthy controls. Repeatability coefficients (RC) for each CMR metric in healthy controls determined the smallest detectable difference between repeated measures.^14^ For cases with CMR abnormalities at baseline, findings were considered: (A) ongoing when CMR metrics were outside reference ranges at follow-up, independently from RC, (B) resolved when change was >RC and CMR metrics were within reference ranges at follow-up. In cases without baseline CMR abnormalities, participants were considered: (A) never affected when CMR was within reference ranges at follow-up, independently from RC, (B) with new onset findings when change was >RC and CMR metrics were outside reference ranges at follow-up. Associations with all exposures were by logistic and linear regression for categoric and continuous dependent variables, respectively. Variables with a significance >0.05 in the univariable models were included in the multivariable analyses. Goodness of fit was performed comparing the actual versus predicted values for an outside validation cohort and doing a visual inspection of residuals of the model. Multivariable stepwise regressions were performed to assess which cardiac metrics at baseline, as continuous variables, were most predictive of poor quality of life, reduced symptom severity and ongoing CMR findings between baseline and follow-up to inform future clinical care.

### Community-delivered diagnostic assessment

Technical success of CMR was determined by reporting quality-assured measures for each variable reported here, and of multiorgan MRI overall, in delivering a report for each patient. For cardiac T1 and T2, technical success was based on value availability for least three AHA segments. Clinical utility of MRI metrics was not directly assessed during the study, as they were used for research only.

## Results

### Characteristics of cardiac abnormalities at 6 months

**Figure 2:**
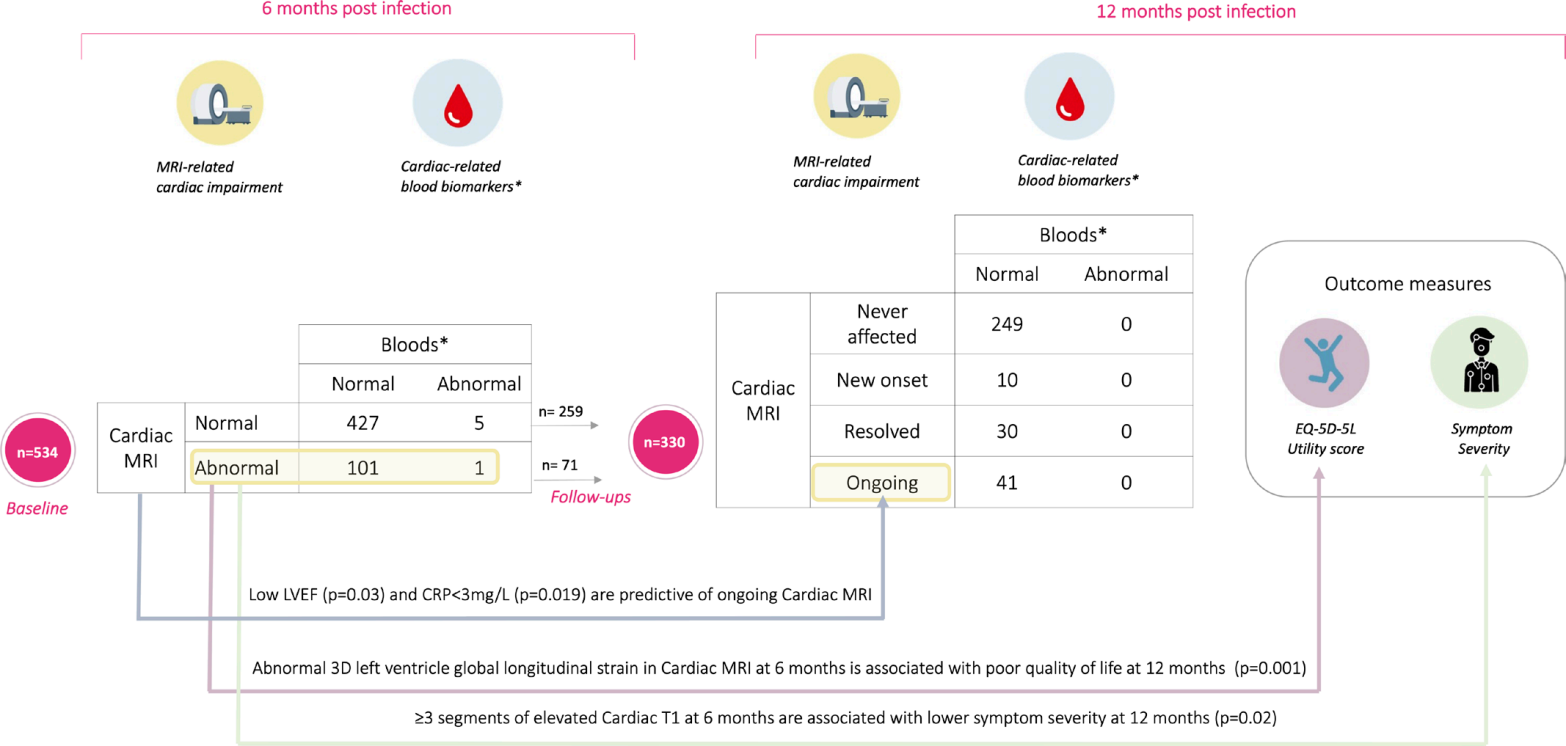
Central illustration. Evolution and characteristics of cardiac abnormalities in Long Covid 1-year post-SARS-CoV-2 infection. Numbers in the table are referring to number of patients. *Referring to high sensitivity cardiac troponin I and B-type natriuretic peptide. CRP, C reactive protein; EQ-5D-5L, EuroQoL-5 dimension-5 level; LVEF, left ventricular ejection fraction.

Of 536 individuals enrolled at baseline, 534 had available CMR data at a median 6 (IQR (4.33–7.26)) months after first COVID-19 symptoms (table 1, figure 1). Of those, 6 (1%) presented with raised cardiac blood biomarkers (high hs-cTnI, n=4 and high NT-proBNP, n=2), but only 1/6 had abnormal CMR with both low LVEF and RVEF at 6 months and acute COVID-19 hospitalisation. However, an additional group of 101 individuals (19%) presented with abnormalities on CMR and normal cardiac blood biomarkers (figure 2, online supplemental tables S2–S4).

**Table 1:** Demographics and characteristics

	6 months							12 months		
	Overall cohort n=534	CMR abnormalities n=102	No CMR abnormalities n=424	P value	CMR abnormalities and hospitalised n=19	CMR abnormalities and non-hospitalised n=83	P value	Ongoing CMR abnormalities n=41	Resolved cardiac function n=30	P value
Demographics										
Age (median (IQR) or mean (SD))	44 (38–52)	43 (37–51)	44 (38–52)	0.41	45 (41–53)	41 (35–51)	0.2	45 (13)	48 (12)	0.22
Sex (% male)	147 (28%)	42 (41%)	103 (24%)	**0.001**	11 (58%)	31 (37%)	0.1	19 (46%)	11 (37%)	0.41
BMI kg/m^2^ (median (IQR))	25.5 (22.6– 29.3)	26.3 (23.1–29.0)	25.3 (22.6–29.4)	0.28	28.0 (23.4–32.0)	26.0 (23.0–28.4)	0.31	25.6 (23.4–28.4)	27.4 (24.5–33.8)	0.09
BMI ≥25 to <30 kg/m^2^ (%)	172 (32%)	38 (37%)	131 (31%)	0.22	7 (37%)	31 (37%)	0.97	15 (37%)	11 (37%)	0.99
BMI ≥30 kg/m^2^ (%)	119 (22%)	23 (23%)	96 (23%)	0.98	6 (32%)	17 (20%)	0.36	8 (20%)	10 (33%)	0.19
Hypertension (%)	44 (8.2%)	12 (12%)	32 (7.5%)	0.17	2 (11%)	10 (12%)	1	5 (12%)	6 (20%)	0.51
Diabetes (%)	10 (1.9%)	3 (2.9%)	7 (1.7%)	0.42	0 (0%)	3 (3.6%)	1	2 (4.9%)	0 (0%)	0.51
Asthma (%)	101 (19%)	22 (22%)	78 (18%)	0.46	4 (21%)	18 (22%)	1	9 (100%)	5 (100%)	1
Previous heart disease	9 (1.7%)	2 (2%)	7 (1.7%)	0.82	0 (0%)	2 (2.4%)	0.49	1 (2.4%)	0 (0%)	0.38
Ethnicity (%): white	475 (89%)	88 (86%)	382 (90%)	0.57	14 (74%)	74 (89%)	0.16	38 (93%)	23 (77%)	**0.02**
Asian	24 (4.5%)	7 (6.9%)	16 (3.8%)		3 (16%)	4 (4.8%)		1 (2.4%)	6 (20%)	
Black	13 (2.4%)	3 (2.9%)	9 (2.1%)		1 (5.3%)	2 (2.4%)		0 (0%)	1 (3.3%)	
Mix	21 (3.9%)	4 (3.9%)	16 (3.8%)	0.15	1 (5.3%)	3 (3.6%)	0.68	2 (4.9%)	0 (0%)	0.9
Other	1 (0.2%)	0 (0%)	1 (0.2%)		0 (0%)	0 (0%)		0 (0%)	0 (0%)	
Smoking status (%): current	348 (65%)	5 (4.9%)	7 (1.7%)		0 (0%)	5 (6.0%)		2 (4.9%)	1 (3.3%)	
Never	13 (2.4%)	66 (65%)	275 (65%)		14 (74%)	52 (63%)		31 (76%)	22 (73%)	
Past	172 (32%)	31 (30%)	141 (33%)		5 (26%)	26 (31%)		8 (20%)	7 (23%)	
Time from first symptom to scan (median (IQR))	182 (132–221)	162 (118–213)	183 (140–223)	**0.05**	141 (77)	173 (72)	0.12	359 (339–394)	380 (323–422)	0.27
Severity										
Hospitalisation at the acute stage (%)	72 (14%)	19 (19%)	51 (12%)	0.08	100 (100%)	0 (0%)	–	7 (17%)	9 (30%)	0.2
Long COVID severity from questionnaires (%):										
Mild	175 (34%)	38 (38%)	135 (33%)	0.38	11 (58%)	27 (33%)	**0.047**	20 (54%)	13 (45%)	0.46
Severe	338 (66%)	62 (62%)	270 (67%)		8 (42%)	54 (67%)		17 (46%)	16 (55%)	
Self-reported symptom severity (%): critical	11 (2.1%)	1 (1.0%)	9 (2.1%)	0.23	1 (5.3%)	0 (0%)	**0.01**	16 (39%)	9 (30%)	0.6
Mild	42 (7.9%)	13 (13%)	29 (6.9%)		0 (0%)	13 (16%)		11 (27%)	7 (23%)	
Moderate	232 (44%)	44 (44%)	186 (44%)		5 (26%)	39 (48%)		14 (34%)	13 (43%)	
Severe	246 (46%)	43 (43%)	198 (47%)		13 (68%)	30 (37%)		0 (0%)	1 (3.3%)	
EQ-5D-5L quality of life (Utility score) (median (IQR))	0.67 (0.49–0.77)	0.66 (0.43–0.77)	0.68 (0.50–0.77)	0.66	0.74 (0.57–0.81)	0.65 (0.42–0.77)	0.08	0.72 (0.55–0.81)	0.71 (0.33–0.84)	0.89
Dyspnoea 12 score (median (IQR))	6 (2–14)	6 (2–12)	7 (2–14)	0.43	4 (2–8)	6 (2–13)	0.52	4 (2–11)	4 (1–11)	0.91
LVD-36 (average, SD)	–	–	–	–	–	–	–	39% (31.3)	36% (28.3)	0.67
Time off work (median (IQR))	56 (14–180)	60 (21–180)	56 (14–180)	0.55	NA	124.8 (129.6)	–	96 (35–270)	135 (40–302)	0.45
Vaccination status (vaccinated at least one dose-%)	10 (1.9%)	2 (2%)	8 (1.9%)	0.96	0 (0%)	2 (2.4%)	0.5	19 (46.3%)	18 (60%)	0.25
Multiorgan impairment										
No organ impairment (%)	227 (43%)	0 (0%)	222 (52%)	**<0.001**	0 (0%)	0 (0%)	1	0 (0%)	16 (53%)	**<0.001**
≥2 organs impaired (%)	118 (22%)	47 (46%)	69 (16%)	**<0.001**	11 (58%)	36 (43%)	0.3	20 (49%)	4 (13%)	**0.002**
≥3 organs impaired (%)	38 (7.1%)	14 (14%)	24 (5.7%)	**0.005**	6 (32%)	8 (9.6%)	**0.02**	5 (12%)	1 (3.3%)	0.39
Symptoms										
No of symptoms (median, IQR)	9^7 11^	10 (8,11)	10 (8,11)	1	10 (8,11.5)	10 (8.5, 11)	0.44	2 (0,5)	4 (0,6)	0.26
Fever (%)	374 (70%)	69 (68%)	299 (71%)	0.62	15 (79%)	54 (66%)	0.27	1 (2.4%)	2 (7%)	0.57
Cough (%)	397 (75%)	81 (80%)	312 (74%)	0.19	16 (84%)	65 (79%)	0.76	2 (4.9%)	9 (30%)	**0.01**
Sore throat (%)	379 (71%)	70 (69%)	302 (72%)	0.65	11 (58%)	59 (72%)	0.23	6 (15%)	5 (17%)	1
Runny nose (%)	175 (33%)	35 (35%)	137 (32%)	0.67	9 (47%)	26 (32%)	0.2	2 (4.9%)	4 (13%)	0.23
Wheezing (%)	268 (50%)	51 (50%)	214 (51%)	0.97	15 (79%)	36 (44%)	**0.01**	4 (9.8%)	1 (3.3%)	0.39
Chest pain (%)	435 (82%)	82 (81%)	347 (82%)	0.81	15 (79%)	67 (82%)	0.75	14 (34%)	12 (40%)	0.61
Muscle aches (%)	472 (89%)	81 (80%)	384 (91%)	**0.002**	14 (74%)	67 (82%)	0.52	13 (32%)	11 (37%)	0.66
Joint pain (%)	394 (74%)	69 (68%)	319 (76%)	0.13	11 (58%)	58 (71%)	0.28	12 (29%)	11 (37%)	0.51
Fatigue or Malaise (%)	522 (98%)	101 (100%)	413 (98%)	0.22	19 (100%)	82 (100%)	1	20 (49%)	19 (63%)	0.22
Shortness of breath (%)	473 (89%)	89 (88%)	377 (89%)	0.72	19 (100%)	70 (85%)	0.12	13 (32%)	16 (53%)	0.07
Inability to walk (%)	177 (33%)	41 (41%)	132 (31%)	0.07	10 (53%)	31 (38%)	0.24	2 (4.9%)	0 (0%)	0.51
Headache (%)	446 (84%)	84 (83%)	356 (84%)	0.77	13 (68%)	71 (87%)	0.08	13 (32%)	10 (33%)	0.89
Seizures (%)	5 (0.9%)	1 (1.0%)	4 (0.9%)	1	0 (0%)	1 (1.2%)	1	0 (0%)	0 (0%)	1
Abdominal pain (%)	285 (54%)	59 (58%)	221 (52%)	0.27	10 (53%)	49 (60%)	0.57	6 (15%)	5 (17%)	1
Diarrhoea (%)	304 (57%)	59 (58%)	242 (57%)	0.85	12 (63%)	47 (57%)	0.64	5 (12%)	5 (17%)	0.73

Characteristics for overall population, CMR abnormalities versus no CMR abnormalities at 6 and 12 months in individuals with Long Covid. Values are presented as mean (SD) and p values calculated with t-test when the data were normally distributed. For variables where data were not normally distributed data are presented with median (IQR) and p values are calculated with Wilcoxon rank sum test. p ≤0.05 are in bold.

BMI, body mass index; CMR, cardiac MR; LVD-36, Left Ventricular Dysfunction Questionnaire.

Demographic differences between groups are presented in table 1, the 102 individuals with CMR findings at 6 months were mostly characterised by reduced LVEF (21/102, 21%) or RVEF (21/102, 21%), low GLS (21/102, 21%) or T1 findings (46/102, 45%) (T1 topographical abnormalities are shown in S5) (table 2). Multiorgan involvement (≥3 organs) was more common in those with CMR abnormalities compared with those without (14% vs 5.7%, p=0.005) (table 1).

**Table 2:** CMR abnormalities in long Covid at 6 and 12 months postinfection

	6 months						12 months		
	CMR abnormalities n=102	No CMR abnormalities n=424	P value	CMR abnormalities and hospitalised, n=19	CMR abnormalities not hospitalised, n=83	P value	Ongoing CMR abnormalities n=41	Resolved CMR abnormalities n=30	P value
Elevated T1	46 (45%)	0 (0%)	**<0.001**	13 (68%)	33 (40%)	**0.02**	13 (32%)	0 (0%)	**<0.001**
Left ventricle									
High end diastolic volume	4 (3.9%)	0 (0%)	**0.001**	1 (5.3%)	3 (3.6%)	0.6	2 (4.9%)	0 (0%)	0.32
High end systolic volume	6 (5.9%)	4 (0.9%)	**0.005**	1 (5.3%)	5 (6.0%)	1	3 (7.3%)	0 (0%)	0.15
Low ejection fraction	21 (21%)	0 (0%)	**<0.001**	5 (26%)	16 (19%)	0.5	9 (22%)	0 (0%)	**0.008**
High stroke volume	1 (1.0%)	3 (0.7%)	0.58	0 (0%)	1 (1.2%)	1	0 (0%)	0 (0%)	1
High ventricular muscle mass	6 (5.9%)	18 (4.2%)	0.44	2 (11%)	4 (4.8%)	0.3	2 (4.9%)	2 (6.7%)	0.60
High ventricular maximum wall thickness	11 (11%)	26 (6.1%)	0.09	3 (17%)	8 (9.6%)	0.4	4 (9.8%)	4 (13%)	0.23
Low global circumferential strain	11 (11%)	13 (3.1%)	**0.002**	2 (11%)	9 (11%)	1	6 (15%)	1 (3.3%)	0.17
Low global longitudinal strain	21 (21%)	0 (0%)	**<0.001**	1 (5.6%)	20 (25%)	0.1	7 (17%)	0 (0%)	**0.02**
Right ventricle									
High end diastolic volume	6 (5.9%)	0 (0%)	**<0.001**	2 (11%)	4 (4.8%)	0.3	3 (7.3%)	0 (0%)	0.15
High end systolic volume	7 (6.9%)	2 (0.5%)	**<0.001**	3 (16%)	4 (4.8%)	0.1	3 (7.3%)	2 (6.7%)	0.79
Low ejection fraction	21 (21%)	0 (0%)	**<0.001**	4 (21%)	17 (20%)	1	12 (29%)	0 (0%)	**<0.001**
High stroke volume	4 (3.9%)	0 (0%)	**0.001**	1 (5.3%)	3 (3.6%)	0.6	2 (4.9%)	0 (0%)	0.32

Prevalence of abnormal CMR findings at 6 and 12 months in individuals with Long Covid.

p ≤0.05 are in bold.

CMR, cardiac MR.

In exploratory analyses, no blood investigations were predictive of CMR abnormalities at 6 months and a full table with prevalence of blood abnormalities and group can be found in online supplemental table S4. At 6 months, 62/102 (62%) individuals with CMR abnormalities presented with severe Long COVID, based on questionnaires (Supplementary methods). Forty-three (43%) and 44 (44%) individuals had severe and moderate symptoms, respectively; most commonly fatigue (100%), shortness of breath (88%), headache (83%), chest pain (81%) and cough (80%). Symptom prevalence was similar regardless of the CMR abnormalities category (table 1).

Follow-up CMR data were available in 330/331 individuals at a median 12.7 (IQR: 11.6–14.3) months since first symptoms; these individuals were all symptomatic at baseline. At 12 months, 51/330 (15%) presented with CMR abnormalities. Of the 102 individuals with CMR abnormalities at 6 months, 71 had follow-up data available (figure 1).

### Resolved CMR abnormalities

At 12 months CMR abnormalities had resolved in 30/71 (42%). At 6 months, CMR in this group showed elevation in T1 (57%), low GLS (21%) and reduced LVEF (20%), with full resolution by 1 year (table 2). By 12 months, 53% had fully resolved multiorgan impairment, and only 1 individual had impairment in ≥3 organs (table 3). Alongside resolution of CMR findings, elevation of NT-proBNP observed at baseline in a single patient of 41 years had resolved by 12 months. No blood investigations were predictive of cardiac recovery (online supplemental table S4).

**Table 3:** Multiorgan impairment (non-cardiac) in individuals at baseline and follow-up

	6 months							12 months		
	Overall cohort n=534	CMR abnormalities n=102	No CMR abnormalities n=424	P value	CMR abnormalities and hospitalised, n=19	CMR abnormalities not hospitalised, n=83	P value	Ongoing CMR abnormalities n=41	Resolved CMR abnormalities n=30	P value
Liver										
cT1 (high)	58 (11%)	13 (13%)	43 (10%)	0.48	3 (16%)	10 (12%)	0.71	6 (15%)	1 (3.4%)	0.23
PDFF (high)	119 (24%)	25 (26%)	92 (24%)	0.62	6 (33%)	19 (25%)	0.55	9 (23%)	5 (17%)	0.56
Volume (high)	35 (6.6%)	6 (5.9%)	29 (6.9%)	0.72	2 (11%)	4 (4.8%)	0.31	3 (7.3%)	3 (10%)	0.69
Kidneys										
Cortex T1 both kidneys (high)	28 (5.3%)	8 (7.8%)	20 (4.8%)	0.21	3 (16%)	5 (6.0%)	0.17	2 (4.9%)	0 (0%)	0.51
Volume, both kidneys (high)	18 (3.4%)	6 (5.9%)	12 (2.9%)	0.14	3 (16%)	3 (3.6%)	0.08	2 (4.9%)	2 (6.9%)	1
Left cortex T1 (high)	61 (12%)	12 (12%)	48 (11%)	0.92	5 (26%)	7 (8.4%)	**0.04**	4 (9.8%)	3 (10%)	1
Right cortex T1 (high)	46 (8.7%)	8 (7.8%)	38 (9.1%)	0.70	3 (16%)	5 (6.0%)	0.17	5 (12%)	1 (3.4%)	0.40
Left volume (high)	28 (5.3%)	7 (6.9%)	21 (5.0%)	0.45	3 (16%)	4 (4.8%)	0.12	2 (4.9%)	2 (6.9%)	1
Right volume (high)	38 (7.2%)	8 (7.8%)	30 (7.1%)	0.81	4 (21%)	4 (4.8%)	**0.04**	4 (9.8%)	2 (6.9%)	1
Pancreas										
T1 (high)	46 (9.1%)	8 (8.4%)	37 (9.2%)	0.81	2 (11%)	6 (7.8%)	0.64	2 (5.9%)	4 (17%)	0.22
PDFF (high)	77 (15%)	14 (14%)	62 (15%)	0.86	4 (22%)	10 (12%)	0.28	4 (11%)	6 (23%)	0.30
Spleen										
Volume (high)	42 (7.9%)	6 (5.9%)	36 (8.6%)	0.37	1 (5.3%)	5 (6.0%)	1	4 (9.8%)	1 (3.4%)	0.39
Length (high)	43 (8.1%)	8 (7.8%)	35 (8.3%)	0.88	1 (5.3%)	7 (8.4%)	1	3 (7.3%)	0 (0%)	0.26
Lungs										
Total deep fractional area change (low)	12 (2.4%)	10 (2.5%)	2 (2.2%)	1	0 (0%)	2 (2.7%)	1	0 (0%)	0 (0%)	1

Prevalence of abnormal CMR findings for multiorgan scans at 6 and 12 months in individuals with Long Covid.

p ≤0.05 are in bold.

CMR, cardiac MR; PDFF, proton-density fat fraction; PDFF, proton-density fat fraction.

Of these individuals, 13/30 (43%) presented with severe Long COVID at baseline, with less symptom burden at follow-up in all but 1 (median 10 and 4 symptoms at 6 and 12 months, respectively) and 5/30 (17%) fully resolving their symptoms (table 1). CMR abnormalities affected quality of life 1 year after infection (mean LVD-36 score 36%) and 13/30 (43%) still presented moderate to severe problems with usual activities. Of 30, 9 (30%) had required acute COVID-19 hospitalisation, and 3 (10%) were hospitalised between 6 and 12 months postinfection.

### Ongoing CMR abnormalities

At 12 months, abnormalities by CMR persisted in 58% (41/71) of individuals. At 6 months, reduced LVEF (p=0.04) and low GLS (p=0.02) were more common, and at 12 months, LVEF, GLS and RVEF were consistently lower (p=0.05, p=0.04 and p=0.04, respectively) (table 4). One individual presented with abnormal T2 imaging at 12 months. Multiorgan impairment was more common in those individuals not resolving their CMR abnormalities (≥2 organs impaired in 49% with ongoing CMR abnormalities, p=0.002) (table 1).

**Table 4:** CMR metrics in those with between ongoing and resolved cardiac abnormalities

		Healthy controls n=92	6 months			12 months		
			CMR abnormalities ongoing at 12 months, n=41	CMR abnormalities resolving at 12 months, n=41	P value	CMR abnormalities ongoing at 12 months, N=41	CMR abnormalities resolving at 12 months, N=30	P value
Global T1 (ms)	1.5T	968 (962, 988)	974 (35)	987 (31)	0.2	982 (26)	970 (36)	0.2
	3T	1172 (1150, 1192)	1196 (37)	1200 (27)	0.8	1200 (1172,1209)	1194 (1188, 1204)	0.9
Left ventricle								
End diastolic volume (mL)		86 (79, 97)	88 (73, 97)	80 (70, 88)	0.18	86 (16)	82 (17)	0.30
End systolic volume (mL)		35 (31, 41)	39 (30, 46)	34 (28, 41)	0.07	37 (10)	33 (8)	0.07
Ejection fraction (%)		59.5 (56.6, 62.7)	55.0 (5.8)	58.1 (6.0)	**0.04**	57.7 (6.0)	60.0 (3.9)	**0.05**
Stroke volume (mL)		52 (46, 58)	45 (40, 53)	46 (43, 50)	0.99	48 (43, 54)	48 (42, 53)	0.96
Ventricular muscle mass (g)		78 (64, 96)	87 (19)	85 (24)	0.72	86 (76, 100)	84 (68, 108)	0.79
Ventricular max wall thickness (mm)		8.91 (8.16, 10.20)	9.45 (8.46, 10.50)	9.24 (8.30, 10.33)	0.50	9.75 (8.77, 10.74)	9.61 (8.64, 10.58)	0.56
Global circumferential strain (%)		−21.28 (2.31)	−19.64 (2.67)	−21.16 (2.44)	**0.02**	−20.43 (2.68)	−21.34 (2.16)	0.13
Global longitudinal strain (%)		−14.68 (−15.95, –13.69)	−12.85 (−14.56, –11.49)	−13.93 (−15.03, –11.89)	0.28	−13.29 (2.59)	−14.49 (2.13)	**0.04**
Right ventricle								
End diastolic volume (mL)		87 (78, 101)	83 (69, 95)	80 (67, 88)	0.52	81 (72, 98)	82 (68, 91)	0.43
End systolic volume (mL)		38 (31, 45)	37 (30, 46)	34 (27, 39)	0.16	36 (29, 43)	34 (24, 40)	0.18
Ejection fraction (%)		57.6 (4.5)	54.9 (5.7)	57.3 (5.5)	0.09	56.1 (6.1)	58.9 (5.1)	**0.04**
High stroke volume (mL)		50 (45, 58)	44 (39, 52)	46 (39, 49)	0.66	46 (40, 53)	46 (41, 50)	0.96

Detailed findings of CMR at 6 and 12 months in individuals with ongoing and resolved cardiac abnormalities. Values are presented as mean (SD) and p values calculated with t-test when the data were normally distributed. For variables where data were not normally distributed data is presented with median (IQR) and p values are calculated with Wilcoxon rank sum test.

p ≤0.05 are in bold.

CMR, cardiac MR.

Symptoms and impact on usual activities as well as quality of life were similar between the ongoing and resolved CMR abnormalities groups. Of 41, 16 (39%) individuals with ongoing CMR abnormalities still presented with severe Long COVID; however, most of them reduced the number of symptoms (median 10 and 2 symptoms at 6 and 12 months, respectively) and 6/41 (15%) patients become asymptomatic (table 1). Of 41, 7 (17%) individuals had acute COVID-19 hospitalisation. Only 1/41 (2%) required hospitalisation between visits. Average time off work was not significantly different between resolved and ongoing impairment groups. Ten individuals with normal cardiac function at 6 months developed CMR abnormalities by 12 months (elevated cardiac T1: n=6, low RVEF: n=4, low LVEF: n=1) (online supplemental table S6).  

### Impact of hospitalisation versus non-hospitalisation in the acute stage and CMR abnormalities

Most individuals (83/102 (81.4%)) with CMR abnormalities did not require hospitalisation at the acute stage. Nevertheless, acute COVID-19 hospitalisation in those with CMR abnormalities (19%) was associated with severe symptoms (68% vs 37%, p=0.01), T1 elevation by CMR (68% vs 40%, p=0.02) and multiorgan involvement (≥3 organs; 32% vs 9.6%, p=0.02), compared with non-hospitalised individuals (tables 1–2).

### Associations of cardiac markers and outcomes in long COVID populations at risk of CMR abnormalities

CMR abnormality at 12 months was mainly predicted by having low LVEF (p=0.03) and CRP levels ≤3 mg/L (p=0.019) at 6 months, based on stepwise multivariable logistic regression. CMR abnormalities as a composite group at 6 months were not predictive of any clinical outcome measures at 12 months; however, low GLS and elevated cardiac T1 at 6 months were predictive of poor quality of life (OR: 0.78 (95% CI 0.67 to 0.91), p=0.001) and lower symptom severity (OR: 0.71 (95% CI 0.52 to 0.96), p=0.02) at 12 months (figure 2).

### Multiorgan MRI (including CMR) and integrated clinical assessment

Technical success of multiorgan MRI was 99.1% and 98.3% at baseline and follow-up assessments, respectively. Technical success of CMR and integrated in-person assessment was 99.6% at first visit and 98.8% at follow-up.

## Discussion

In the largest community-based study to-date with cardiac MR follow-up over 1 year in a mainly non-hospitalised, post-COVID-19 cohort with little prior cardiac disease, we report three new findings. First, CMR abnormalities were common (one in five individuals at 6 months) and commonly persisted (three out of five individuals at 12 months). Second, CMR abnormalities were found even without acute COVID hospitalisation (83/462, 18%). Third, cardiac blood biomarkers and symptoms were not predictive of composite CMR abnormalities but abnormal individual CMR parameters (eg, LVEF, 3D global longitudinal strain and cardiac T1) were associated with ongoing CMR findings, lower quality of life or reduced symptom severity at 12 months.

### Characteristics and trajectory of cardiac abnormalities

Our results indicate that, despite women being more affected by Long COVID, men have higher risk of cardiac abnormalities.^15^ Potential contributory factors include: influence of biological sex on expression and regulation of ACE 2, sex differences in genetic and hormonal regulation of immune responses,^16^ sex-dependent patterns of coagulation, smoking or drinking.^4 5 17 18^

Published CMR studies in Long COVID vary by study design, cohort, follow-up duration, definition of cardiac abnormalities and estimated prevalence of cardiac abnormalities (26%–60%).^6 11 19–23^ A recent review^9^ highlighted under-representation of affected individuals from community-based settings, especially monitoring non-hospitalised individuals over time, which we address in this study. When COVID-19-related and classical myocardial injury are compared,^8^ only 9% of individuals fulfil acute myocarditis criteria and those with more severe disease are more likely to exhibit chronic inflammation and impaired cardiac function. We report prevalence of CMR abnormalities (19% and 15% at 6 and 12 months) consistent with previous studies, providing standardisation of metrics and definition, which can be used at scale in research and practice to document and monitor cardiac abnormalities.^6 11 16 19 20^ We confirm that abnormalities in T1 (in line with previous research,^6 9–11 19 22^ T2 and LGE, as well as functional abnormalities,^5 11 23 24^ are most common in Long COVID patients. Acute COVID can present with myocardial inflammation; ongoing COVID-19 patients can also have myocarditis, but it is harder to diagnose, and often missed with echocardiography. More pertinently, the observed functional changes may be due to inflammation and other aetiologies (eg, pulmonary disease, microinfarctions, metabolic dysregulation), and further mechanistic work is required to explore associations with CMR markers seen here.

In 58 hospitalised individuals, 3 months post-COVID-19, there were persistent abnormalities in cardiac T1 (26%) and multiple organs (eg, 29% with increased cortical T1, a marker of kidney inflammation). At 6 months, 52% had persistent symptoms and CMR abnormalities.^19^ In the first 201 individuals in our study, we observed multiorgan impairment (29%; cardiac: 26%; renal: 4%).^11^ In 443 individuals, 10 months after mild-to-moderate COVID-19, subclinical multiorgan impairment was associated with CMR abnormalities (reduced left and right ventricular systolic function).^10^ At 12 months, the longest follow-up duration to-date, we confirm 54% of individuals with CMR abnormalities do not fully recover.

### Impact of acute hospitalisation for COVID-19 on cardiac abnormalities

Most individuals presenting with CMR abnormalities at baseline did not require acute COVID-19 hospitalisation (81%). One individual with elevated cardiac-related blood biomarkers had CMR abnormalities at 6 months and acute COVID-19 hospitalisation. Blood biomarkers and symptoms did not differentiate hospitalised and non-hospitalised groups. On MRI, cardiac T1 abnormalities^4 25^ and multiorgan involvement (particularly renal)^5 11 19^ were more prevalent in those with CMR abnormalities and acute COVID-19 hospitalisation, as in other published studies.^8 13 26 27^

### Clinical management pathways in Long COVID populations at risk of cardiac abnormalities

Cardiac-related blood biomarkers may be raised in early convalescence from COVID-19,^28^ but did not aid detection of CMR abnormalities in Long COVID in our study, despite 19% having CMR abnormalities, supported by other research.^5 23 24^ Burden and improvement in symptoms 6 months after COVID-19 were neither correlated with resolution on CMR nor lung parenchymal recovery.^5^ Early MRI assessment may identify organ-specific impairment (including cardiac), leading to early referral for appropriate specialist assessment and treatment, in contrast to the experience of many patients who are currently having multiple appointments with multiple specialists for multiple assessments. In a cluster-randomised design, the STIMULATE-ICP trial is currently evaluating whether multiorgan MRI (Coverscan) can aid diagnosis and follow-up of cardiac and multiorgan impairment in Long COVID, and reduce burden to healthcare systems, already struggling due to COVID-19-related lack of resources and backlogs, while achieving integrated care.^29^

Cardiac findings could inform design of Long COVID treatment algorithms. Abnormal GLS is associated with cardiac remodelling (indicative of more severe cardiac disease),^26^ and predictive of low quality of life at 12 months. Elevated T1 was predictive of lower symptom severity at 12 months. There may be multiple cardiac subgroups in Long COVID, potentially detected by CMR early postinfection. These subtypes may be related to pulmonary hypertension,^13^ pre-existing comorbidities^27^ and post-COVID-19 myocardial inflammation,^8^ but require further study and validation.

Comprehensive multiorgan MRI assessment may help clinical decision making and improve healthcare access and provision. Evidence of cardiac involvement could guide follow-up assessment and identification of Long COVID subtypes in research and practice. Interventional trials with prespecified subgroup analysis and improved definitions of cardiac abnormality (not only myocarditis centred), are required to inform cost-effective therapies.

### Strengths and limitations

This is the largest longitudinal study to-date of cardiac abnormality in Long COVID with detailed biochemical and imaging characterisation of multiorgan function starting in April 2020. We included healthy, age-matched controls. All MRI was non-contrast. We recruited a real-world cohort at lower risk of COVID-19 severity and mortality. Unlike other studies,^30^ our approach offers quick, scalable assessment using standard MRI scanners. There are limitations. First, our CMR protocol excluded gadolinium contrast, the main reason for this was to reduce the scanning times, contact-time between the patient and the healthcare worker, and to avoid potential renal complications related to COVID-19. This was backed by previous research, supporting the use of native non-invasive T1 mapping to characterise myocardial inflammation,^26^ and did not have sufficient statistical power in cardiac T2 collection, relying on native non-invasive T1 mapping to characterise myocardial inflammation, validated for acute myocarditis.^31^ Second, we are not able to define whether these individuals presented with multiorgan abnormalities before their COVID-19 infection, although clinical diagnoses were recorded. Third, we did not have follow-up scans on individuals without impairment at baseline and a third of patients with CMR abnormalities at baseline withdrew or were lost to follow-up. Fourth, we did not have pre-COVID-19 cardiac or multiorgan imaging available in participants. Fifth, our study population was not ethnically diverse, and COVID-19 has disproportionately affected non-white individuals. In addition, our study recruited patients during the first wave of the pandemic, when testing was not broadly available, mainly via patient support groups rather than a systematic screen of post-COVID-19 patients, as Long COVID clinics were only set up at the end of our recruitment and this may represent a bias.

## Conclusion

CMR shows that cardiac abnormality persists in Long COVID in some individuals up to 12 months after first symptoms. CMR abnormalities (left ventricular or right ventricular dysfunction/dilatation and/or abnormal T1mapping), are associated with acute COVID-19 hospitalisation and male gender, but subtypes of disease (based on symptoms, examination and investigations) are yet to be established. Therapeutic options and effective clinical pathways require urgent clinical trials.

## Data Availability

All data relevant to the study are included in the article or uploaded as online supplemental information.
