# Supplementary material for: Cardiac abnormalities in Long COVID 1-year post-SARS-CoV-2 infection
Source: Open Heart. 2023 Feb 23;10(1):e002241. doi: 10.1136/openhrt-2022-002241 (PMC9950586; doi:10.1136/openhrt-2022-002241)
Supplement: Supplementary data [file openhrt-2022-002241supp001.pdf]

Cardiac abnormalities in Long Covid 1-year post-SARS-CoV-2 infection.

Supplementary materials

Page Number	Contents
2	Supplementary methods
4	Supplementary results
6	Table S1: Reference Ranges for imaging metrics across organs
9	Table S2: Demographics of HC compared to the Long COVID cohort, with and without CMR abnormalities
11	Table S3: Differences in CMR abnormalities and symptoms by COVID-19 diagnosis method
12	Table S4: Blood investigations by abnormalities on CMR
20	Figure S5: T1 topographical abnormalities in patients classified with CMR abnormalities
21	Table S6. Detailed CMR findings in new onset cardiac abnormalities at 12 months
22	Supplementary References

Supplementary methods

1. **Study recruitment:** The study website (coverscan.com) was advertised by Perspectum or by iWGC (an independent platform and service provider for patient experience data collection and analysis) via online posting (social media, website banners) and by global invitation emails to applicable charities and consented patient networks. Participants interested in the study registered their interest by responding to a link located in the study's webpage. Participant response overwhelmed capacity so we randomly selected participants to be contacted for phone screening interview, without a formal algorithm. Participation was voluntary (no remuneration).

2. **Classification of Severity:** Long COVID disease severity categories are described below (a, b).

   a. **Definition Long COVID, based on validated questionnaires** (1–3):

	Dyspnoea 12 score (1)		EQ-5D-5L usual activity score*(4)
Severe Long COVID	≥10	or	≥3
Mild Long COVID	Not fulfilling neither of the conditions above		

*** EQ-5D-5L score is based on the scoring in the following question:**
"Please tick the ONE box that best describes your health TODAY"
USUAL ACTIVITIES (e.g., work, study, housework, family, or leisure activities)
- *I have no problems doing my usual activities (score 1)*
- *I have slight problems doing my usual activities (score 2)*
- *I have moderate problems doing my usual activities (score 3)*
- *I have severe problems doing my usual activities (score 4)*
- *I am unable to do my usual activities (score 5)*

   b. **Definition of symptom severity, based on self-reported perceived severity:**

Patient asked to select one of the following options:
Critical acute respiratory distress syndrome (ARDS)
Severe disease
Moderate disease
Mild disease
Asymptomatic disease

   c. **Severe breathlessness**
   Score of ≥10 in the Dyspnoea-12 validated questionnaire

3. **List of blood biomarkers assessed in this study:**

Haemoglobin, HCT, red cell count, MCV, MCH , MCHC, RDW , platelet count, MPV, white cell count , neutrophils, lymphocytes monocytes, eosinophils, basophils, ESR, sodium, potassium, chloride, bicarbonate, urea, creatinine, bilirubin, alkaline phosphatase, aspartate transferase , alanine transferase , LDH , CK , gamma , total protein , albumin , globulin, calcium, magnesium, phosphate, uric acid, triglycerides, fasting triglycerides, cholesterol, fasting cholesterol, HDL cholesterol, LDL cholesterol, iron, TIBC, transferrin saturation, CRP high

Roca-Fernandez A, *et al. Open Heart* 2023; 10:e002241. doi: 10.1136/openhrt-2022-002241

sensitivity, troponin I high sensitivity, amylase, ferritin, lipase, thyroid stimulating hormone, testosterone, insulin , C-peptide, NTpro-BNP.

4. Imaging acquisitions:

- Cardiac imaging involved a combination of several gated cine series, two long axis cines (horizontal long axis – HLA and vertical long axis – VLA), and a complete short axis stack covering the left ventricle (LV) and right ventricle (RV). This acquisition mirrors the one used at the UK Biobank and is a standardized approach (5). Three short-axis were acquired at the basal, mid, and apical levels of the left ventricle, for T1 mapping using MOLLI, and for T2 mapping using a T2 preparation pulse applied with different T2 preparation times, to impart T2 signal contrast, and a subsequent readout is performed by using a steady-state free precession (SSFP) or a fast low angle shot (FLASH). Cardiac T2 was only collected at the tail end of recruitment and therefore there was insufficient sample size (27/534 at baseline) for inclusion in manuscript results. In line with recent research in amyloidosis and published consensus on myocardial inflammation (6)(7), late gadolinium enhancement was not undertaken in this study where a combination of speed, cost, convenience, and hepatotoxicity avoidance were required to deliver CMR at scale.

Cardiac mapping	
T1	
1.5T Siemens	Modified Look-Locker inversion recovery sequence (MOLLI): For HR >700 ms/beat, TR= ~312.64 ms, TE= ~1.33 or Min ms, Flip angle= 35°, FOV= 384 mm,SL= 8mm, Ny*Nx= 256x256 matrix, Inversion Scheme= 5(3)3, parallel imaging technique/factor= GRAPPA2, BW= 1085 Hz/pixel. For HR <700 ms/beat, TR= ~312.64 ms, TE= ~1.33 or Min ms, Flip angle= 35°, FOV= 384 mm,SL= 8mm, Ny*Nx= 192 x 192 matrix, Inversion Scheme= 5(3)3, parallel imaging technique/factor= GRAPPA2, BW= 1085 Hz/pixel.
3T Siemens	MOLLI: For HR >700 ms/beat,TR= ~ 280.56 ms, TE= ~ 1.12 or Min ms, Flip angle= 35°, FOV= 360 mm,SL= 8mm, Ny*Nx= 256x256 matrix, Inversion Scheme= 5(3)3, parallel imaging technique/factor= GRAPPA2, BW= 1085 Hz/pixel. For HR <700 ms/beat, TR= ~ 272.13ms, TE= ~ 1.2 or Min ms, Flip angle= 35°, FOV= 360 mm,SL= 8mm, Ny*Nx= 192 x192 matrix, Inversion Scheme= 5(3)3, parallel imaging technique/factor= GRAPPA2, BW= 1085 Hz/pixel.
T2	
1.5T Siemens	2D SSFP sequence (TrueFISP): TR= ~ 214.07 ms or Min, TE= ~ 1.23 ms, FOV= 384x308 mm^2, SL= 8mm, Ny*Nx= 192x116 matrix, segment =58, parallel imaging technique/factor= GRAPPA2, BW= 1185 Hz/pixel
3T Siemens	2D TurboFLASH sequence: TR= ~ 214.07 ms or Min, TE= Min, FOV= 360x288 mm^2, SL= 8mm, Ny*Nx= 192x116 matrix, segment = 58, parallel imaging technique/factor= GRAPPA2, BW= 1185 Hz/pixel.

- Liver and pancreas imaging used the LiverMultiScan acquisition protocol (Perspectum, Oxford, UK), which involves 3 single 2D axial slice breath-held acquisitions that

separately are sensitive to the fat content (proton density fat fraction, or PDFF), to T2* (which is representative of liver iron content) and a MOLLI-T1 measurement (providing a measurement of tissue water), additionally a volumetric scan was used that covers the entire liver (8).

- Lungs: Two dynamic cine MR acquisitions were acquired in the coronal plane with a 306.91ms temporal resolution: one 40 s acquisition with the patient instructed to breathe normally and a second 30 s acquisition with the patient instructed to breathe deeply.
- Kidney: single coronal view that was able to image both kidneys. Imaging contrasts were MOLLI-T1, and a spoiled gradient recalled acquisition (SPGR).
- Spleen: Volumetric SPGR MRI images

5. Image Analysis:

- Cardiac: Experienced cardiac MRI analysts used CVI42v5.11 (Cardiovascular Imaging Inc, Canada) to trace manually the myocardium in the end-diastolic and end-systolic phases in each of the short-axis views, following the standard UK Biobank evaluation approach as previously described (9). We reported ventricular function; end systolic and diastolic volume; stroke volume and ejection fraction in both ventricles; left ventricular muscle mass and ventricular max wall thickness and global longitudinal and circumferential 3D strain metrics. Mean Cardiac T1 and T2 were determined for each of the 16 cardiac segments (of the AHA 17 segment model excluding the apex)(10).
- Liver Images were analysed by data analysts experienced at using the LiverMultiScan (Perspectum, Oxford, UK) software. This yielded global metrics in each liver of PDFF (proton density fat fraction), T2*, and cT1 (cT1 is a measurement of T1 that has been corrected for the confounding effects of iron and standardised to 3 Tesla; it is elevated with disease).
- Pancreas images were analysed in an equivalent manner to the above except the software used was not FDA-cleared and iron correction was not performed. The output T1 was standardized to 3 Tesla.
- Lung cine imaging allowed the measurement of the area of the left and right lungs through the breathing cycle in the coronal plane, which used automated methods that were reviewed by image analysts. The periodicity of the area fluctuations was used to determine the respiratory rate. All analysis was performed in-house using MATLAB based tools. The method was validated by measuring the correlation between the change in area and the forced vital capacity, the latter being measured using spirometry. Patient respiration was assessed by imaging a single 2D coronal slice of the lungs over 30 seconds using a dynamic cine MRI acquisition, during which the patient instructed to breathe deeply.
- Kidney: assessed using in-house tools to fit parametric maps and to allow trained analysts to make measurements. The kidney cortex was manually segmented using the MOLLI-T1 map to guide the boundary. Multiple regions-of-interests were manually placed within the cortex to extract a median value of cortical T1 in each kidney. Volumetric delineations of the kidneys were derived from SPGR MRI images. Automated delineations were produced using a 3D convolutional neural network, trained on expert annotations. Delineations were manually checked, and corrected, if necessary, for each subject. In addition to kidney cortex T1 and kidney volume, we also derived kidney length measurements, in the inferior-superior axis, from the same organ

segmentations and assessed the correlation of kidney length and kidney volume measurements

- Spleen: Volumetric delineations were derived from SPGR MRI images. Automated delineations were produced using a 3D convolutional neural network, trained on expert annotations. Delineations were manually checked, and corrected, if necessary, for each subject.
- Organ abnormality: Calculated for each organ based on evidence of any of the measurements appearing out of reference range (*Liver:* elevated cT1 or Fat; *Kidney:* elevated T1 or volume; *Pancreas:* elevated sT1 or Fat; *Heart:* elevated T1 in 3 or more segments, decreased RV or LV EF or increased LV or RV EDV or increased LV global longitudinal strain; *Spleen*: elevated volume; *Lung:* reduced fractional area volume). Single organ impairment was based on ≥1 organ impairment and multi-organ impairment was based on ≥2 organ impairments.

6. Reference Ranges for imaging markers:

All values but organ volumes were calculated using sex and age matched HC (n=92) (Healthy Controls) scanned at 1.5T and 3T for this study calculating 2.5% (lower threshold) and 97.5% percentiles (upper threshold). Organ volumes were calculated from a combined cohort of the 92 healthy controls and 1744 BMI matched participants (N=1836 from N=36) from the UK Biobank, (11), representing all sex and height subgroups, as these are known confounders of organ size.(12) (*) Reference ranges for the liver cT1 and liver PDFF have been taken from the available literature (13), as the LMS technology have been widely used and tested in multiple clinical trials and research settings. For pancreas PDFF, which has a positive skew in the distribution, reference ranges were extracted with the 95% percentile. ($) T2 repeatability coefficients are not provided as this metric was only really available for follow up patients. (§) Right and left cortical T1 limits were averaged for analysis (**Table S1**).

Roca-Fernandez A, *et al. Open Heart* 2023; 10:e002241. doi: 10.1136/openhrt-2022-002241

Table S1: Reference Ranges for imaging metrics across organs

	Gender	Field Strength	Height (cm)	Lower threshold	Upper threshold (*)	Repeatability coefficient
CARDIAC METRICS						
Field strength independent variables (BSA corrected)						
Left end diastolic volume (mL)	F	-	-	-	108	17
Left end diastolic volume (mL)	M	-	-	-	132	17
Right end diastolic volume (mL)	F	-	-	-	110	19
Right end diastolic volume (mL)	M	-	-	-	139	19
Left end Systolic volume (mL)	F	-	-	-	47	12.5
Left end Systolic volume (mL)	M	-	-	-	57	12.5
Right end Systolic volume (mL)	F	-	-	-	49	12
Right end Systolic volume (mL)	M	-	-	-	60	12
Left Stroke volume (mL)	F	-	-	-	66	16
Left Stroke volume (mL)	M	-	-	-	84	16
Right Stroke volume (mL)	F	-	-	-	65	16
Right Stroke volume (mL)	M	-	-	-	84	16
Field strength independent variables (non-BSA corrected)						
Global circumferential strain 3D (%)	F	-	-	-	-18.1	2.5
Global circumferential strain 3D (%)	M	-	-	-	-16.8	2.5
Global longitudinal strain 3D (%)	F	-	-	-	-11.5	5.1
Global longitudinal strain 3D (%)	M	-	-	-	-7.8	5.1
Left ventricle ejection fraction (%)	F	-	-	52	-	6.6
Left ventricle ejection fraction (%)	M	-	-	51	-	6.6
Right ventricle ejection fraction (%)	F	-	-	50	-	7.0
Right ventricle ejection fraction (%)	M	-	-	50	-	7.0
Left ventricular max wall thickness (mm)	F	-	-	-	10.6	2.1
Left ventricular max wall thickness (mm)	M	-	-	-	14	2.1
Left ventricular muscle mass (g)	F	-	-	-	95	13
Left ventricular muscle mass (g)	M	-	-	-	151	13
Field strength Dependent variables: 1.5T						
Global T1 ref range (ms)	F	1.5T	-	-	1042	-
Global T1 ref range (ms)	M	1.5T	-	-	997	-
Segment 1: T1 basal anterior (ms)	F	1.5T	-	-	1043	42
Segment 1: T1 basal anterior (ms)	M	1.5T	-	-	1000	42
Segment 2: T1 basal anteroseptal (ms)	F	1.5T	-	-	1031	49
Segment 2: T1 basal anteroseptal (ms)	M	1.5T	-	-	1022	49
Segment 3: T1 basal inferoseptal (ms)	F	1.5T	-	-	1031	54
Segment 3: T1 basal inferoseptal (ms)	M	1.5T	-	-	1001	54
Segment 4: T1 basal inferior (ms)	F	1.5T	-	-	1091	57
Segment 4: T1 basal inferior (ms)	M	1.5T	-	-	995	57
Segment 5: T1 basal inferolateral (ms)	F	1.5T	-	-	1042	55
Segment 5: T1 basal inferolateral (ms)	M	1.5T	-	-	998	55
Segment 6: T1 basal anterolateral (ms)	F	1.5T	-	-	1041	54
Segment 6: T1 basal anterolateral (ms)	M	1.5T	-	-	979	54
Segment 7: T1 mid anterior (ms)	F	1.5T	-	-	1014	52
Segment 7: T1 mid anterior (ms)	M	1.5T	-	-	969	52
Segment 8: T1 mid anteroseptal (ms)	F	1.5T	-	-	1030	39
Segment 8: T1 mid anteroseptal (ms)	M	1.5T	-	-	1006	39
Segment 9: T1 mid inferoseptal (ms)	F	1.5T	-	-	1036	37
Segment 9: T1 mid inferoseptal (ms)	M	1.5T	-	-	994	37
Segment 10: T1 mid inferior (ms)	F	1.5T	-	-	1035	44
Segment 10: T1 mid inferior (ms)	M	1.5T	-	-	1023	44
Segment 11: T1 mid inferolateral (ms)	F	1.5T	-	-	1016	44
Segment 11: T1 mid inferolateral (ms)	M	1.5T	-	-	982	44
Segment 12: T1 mid anterolateral (ms)	F	1.5T	-	-	1029	62
Segment 12: T1 mid anterolateral (ms)	M	1.5T	-	-	979	62
Segment 13: T1 apical anterior (ms)	F	1.5T	-	-	1059	86
Segment 13: T1 apical anterior (ms)	M	1.5T	-	-	1004	86
Segment 14: T1 apical septal (ms)	F	1.5T	-	-	1065	48
Segment 14: T1 apical septal (ms)	M	1.5T	-	-	992	48
Segment 15: T1 apical inferior (ms)	F	1.5T	-	-	1070	43
Segment 15: T1 apical inferior (ms)	M	1.5T	-	-	1003	43
Segment 16: T1 apical lateral (ms)	F	1.5T	-	-	1040	70
Segment 16: T1 apical lateral (ms)	M	1.5T	-	-	1011	70
Global T2 ref range (ms) ($)	-	1.5T	-	-	51	-
Field strength Dependent variables: 3T						
Global T1 ref range (ms)	F	3T	-	-	1255	-
Global T1 ref range (ms)	M	3T	-	-	1214	-

6

	Gender	Field Strength	Height (cm)	Lower threshold	Upper threshold (*)	Repeatability coefficient
Segment 1: T1 basal anterior (ms)	F	3T	-	-	1226	72
Segment 1: T1 basal anterior (ms)	M	3T	-	-	1201	72
Segment 2: T1 basal anteroseptal (ms)	F	3T	-	-	1248	70
Segment 2: T1 basal anteroseptal (ms)	M	3T	-	-	1218	70
Segment 3: T1 basal inferoseptal (ms)	F	3T	-	-	1251	74
Segment 3: T1 basal inferoseptal (ms)	M	3T	-	-	1218	74
Segment 4: T1 basal inferior (ms)	F	3T	-	-	1271	112
Segment 4: T1 basal inferior (ms)	M	3T	-	-	1231	112
Segment 5: T1 basal inferolateral (ms)	F	3T	-	-	1240	109
Segment 5: T1 basal inferolateral (ms)	M	3T	-	-	1209	109
Segment 6: T1 basal anterolateral (ms)	F	3T	-	-	1200	61
Segment 6: T1 basal anterolateral (ms)	M	3T	-	-	1193	61
Segment 7: T1 mid anterior (ms)	F	3T	-	-	1266	90
Segment 7: T1 mid anterior (ms)	M	3T	-	-	1161	90
Segment 8: T1 mid anteroseptal (ms)	F	3T	-	-	1264	89
Segment 8: T1 mid anteroseptal (ms)	M	3T	-	-	1219	89
Segment 9: T1 mid inferoseptal (ms)	F	3T	-	-	1272	74
Segment 9: T1 mid inferoseptal (ms)	M	3T	-	-	1226	74
Segment 10: T1 mid inferior (ms)	F	3T	-	-	1279	84
Segment 10: T1 mid inferior (ms)	M	3T	-	-	1228	84
Segment 11: T1 mid inferolateral (ms)	F	3T	-	-	1226	60
Segment 11: T1 mid inferolateral (ms)	M	3T	-	-	1210	60
Segment 12: T1 mid anterolateral (ms)	F	3T	-	-	1278	75
Segment 12: T1 mid anterolateral (ms)	M	3T	-	-	1228	75
Segment 13: T1 apical anterior (ms)	F	3T	-	-	1271	63
Segment 13: T1 apical anterior (ms)	M	3T	-	-	1227	63
Segment 14: T1 apical septal (ms)	F	3T	-	-	1280	62
Segment 14: T1 apical septal (ms)	M	3T	-	-	1230	62
Segment 15: T1 apical inferior (ms)	F	3T	-	-	1257	57
Segment 15: T1 apical inferior (ms)	M	3T	-	-	1202	57
Segment 16: T1 apical lateral (ms)	F	3T	-	-	1254	77
Segment 16: T1 apical lateral (ms)	M	3T	-	-	1214	77
Global T2 ref range (ms) ($)	-	3T	-	-	46	-
LIVER METRICS						
Field strength independent variables						
cT1 ROI (ms)	-	-	-	-	800 (*)	48
PDFF %	-	-	-	-	5 (*)	1.5
Volume (mL)	F	-	<164	-	1778	64
Volume (mL)	M	-	<164	-	2003	64
Volume (mL)	F	-	≥ 164, < 250	-	2049	64
Volume (mL)	M	-	≥ 164, < 250	-	2284	64
KIDNEY METRICS						
Field strength independent variables						
Left Volume (mL)	F	-	<164	-	177	10
Left Volume (mL)	M	-	<164	-	221	10
Left Volume (mL)	F	-	≥ 164, < 250	-	192.	10
Left Volume (mL)	M	-	≥ 164, < 250	-	255	10
Right Volume (mL)	F	-	<164	-	176	8
Right Volume (mL)	M	-	<164	-	207	8
Right Volume (mL)	F	-	≥ 164, < 250	-	186	8
Right Volume (mL)	M	-	≥ 164, < 250	-	229	8
Field strength Dependent variables: 1.5T						
Cortex T1 (ms) (§)	-	1.5T	-	-	1154	76
Field strength Dependent variables: 3T						
Cortex T1 (ms) (§)	-	3T	-	-	1512	68
PANCREAS						
Field strength independent variables						
sT1 ROI (ms)	-	-	-	-	821	74
PDFF %	-	-	-	-	6.6 (*)	2.8
SPLEEN						
Field strength independent variables						
Volume (mL)	F	-	<164	-	254	17
Volume (mL)	M	-	<164	-	392	17
Volume (mL)	F	-	≥ 164, < 250	-	293	17
Volume (mL)	M	-	≥ 164, < 250	-	411	17.
LUNG						
Field strength independent variables						

	Gender	Field Strength	Height (cm)	Lower threshold	Upper threshold (*)	Repeatability coefficient
Total deep fractional area change (%)	-	-	-	22	-	15.9

Table S2: Demographics of HC compared to the Long COVID cohort, with and without CMR abnormalities

Demographic	HC, N = 91	COVID, N = 534	Post-COVID CMR abnormalities n=102	Post-COVID No CMR abnormalities n=424	P (HC vs COVID cohort)	P (HC vs Post-COVID CMR abnormalities)	P (HC vs Post-COVID No CMR abnormalities)
Age	44 (33, 53)	44 (38, 52)	43 (37, 51)	44 (38, 52)	0.6	>0.9	0.5
Sex (% male)	30 (33%)	147 (28%)	42 (41%)	103 (24%)	0.3	0.2	0.086
BMI kg/m^2 (Median (IQR))	22.8 (20.9, 25.1)	25.5 (22.6, 29.3)	26.3 (23.1, 29.0)	25.3 (22.6, 29.4)	**<0.001**	<0.001	<0.001
BMI ≥25 to <30 kg/m^2 (%)	20 (22%)	172 (32%)	38 (37%)	131 (31%)	**0.051**	**0.021**	0.090
BMI ≥30 kg/m^2 (%)	3 (3.3%)	119 (22%)	23 (23%)	96 (23%)	**<0.001**	**<0.001**	**<0.001**
Ethnicity (%)					0.3	0.3	0.3
White	84 (92%)	475 (89%)	88 (86%)	382 (90%)			
Asian	6 (6.6%)	24 (4.5%)	7 (6.9%)	16 (3.8%)			
Black	0 (0%)	13 (2.4%)	3 (2.9%)	9 (2.1%)			
Mix	1 (1.1%)	21 (3.9%)	4 (3.9%)	16 (3.8%)			
Other	0 (0%)	1 (0.2%)	0 (0%)	1 (0.2%)			
Smoking					**0.02**	**0.013**	**0.01**
Never	71 (78%)	348 (65%)	66 (65%)	275 (65%)			
Current	3 (3.3%)	13 (2.4%)	5 (4.9%)	7 (1.7%)			
Past	17 (19%)	172 (32%)	31 (30%)	141 (33%)			
Hypertension	0 (0%)	44 (8.2%)	12 (12%)	32 (7.5%)	**0.005**	**<0.001**	**0.007**

Roca-Fernandez A, *et al. Open Heart* 2023; 10:e002241. doi: 10.1136/openhrt-2022-002241

Diabetes	0 (0%)	10 (1.9%)	3 (2.9%)	7 (1.7%)	0.4	0.20	0.60
Asthma	2 (2.2%)	101 (19%)	22 (22%)	78 (18%)	**<0.001**	**<0.001**	**<0.001**
Previous heart disease	0 (0%)	9 (1.7%)	2 (2%)	7 (1.7%)	**0.21**	**0.5**	**0.6**

Table S3: Differences in CMR abnormalities and symptoms by COVID-19 diagnosis method

	Clinically diagnosed COVID-19	COVID-19 positive PCR test result	Proportion test P value
Prevalence of CMR abnormalities at baseline			
CMR abnormalities	43 (18%)	59 (20%)	0.557
No CMR abnormalities	196 (82%)	236 (80%)	0.557
Total	239	295	
Severity of symptoms at baseline			
Mild/moderate	113 (47%)	161 (55%)	0.138
Severe/Extreme	125 (52%)	132(45%)	0.138
Total	238	293	
Trajectory of CMR abnormalities at 12 months			
Never	117(80%)	132 (72%)	0.08
New	3 (2%)	7 (4%)	0.35
Ongoing	13 (9%)	28 (15%)	0.09
Resolved	13 (9%)	17 (9%)	0.92
Total	146	184	-

Table S4: Blood investigations by abnormalities on CMR

	6 months										12 months		
	COVID, N=534	No CMR abnormalities, N = 424	CMR abnormalities, N = 102	P	CMR abnormalities hospitalised, N = 19	CMR abnormalities non hospitalized, N = 83	P	Ongoing CMR abnormalities, N = 41	Resolved CMR abnormalities, N = 30	P	Ongoing CMR abnormalities, N = 41	Resolved CMR abnormalities, N = 30	P
**Haemoglobin**													
H	6 (1.2%)	4 (1.0%)	2 (2.2%)	0.36	0 (0%)	2 (2.6%)	0.60	1 (2.6%)	0 (0%)	0.51	1 (2.4%)	0 (0%)	1.00
L	13 (2.6%)	9 (2.2%)	3 (3.3%)		1 (6.7%)	2 (2.6%)		2 (5.3%)	0 (0%)		1 (2.4%)	0 (0%)	
N	487 (96%)	393 (97%)	87 (95%)		14 (93%)	73 (95%)		35 (92%)	27 (100%)		39 (95%)	27 (100%)	
Missing	28	18	10		4	6		3	3		0	3	
**HCT**													
H	10 (2.0%)	9 (2.2%)	1 (1.1%)	0.69	0 (0%)	1 (1.3%)	0.42	1 (2.6%)	0 (0%)	1.00	1 (2.4%)	2 (7.4%)	0.56
L	8 (1.6%)	5 (1.2%)	2 (2.2%)		1 (6.7%)	1 (1.3%)		1 (2.6%)	0 (0%)		0 (0%)	0 (0%)	
N	488 (96%)	392 (97%)	89 (97%)		14 (93%)	75 (97%)		36 (95%)	27 (100%)		40 (98%)	25 (93%)	
Missing	28	18	10		4	6		3	3		0	3	
**Red cell count**													
H	14 (2.8%)	13 (3.2%)	1 (1.1%)	0.69	0 (0%)	1 (1.3%)	0.52	0 (0%)	0 (0%)	0.51	1 (2.4%)	1 (3.7%)	0.38
L	17 (3.4%)	12 (3.0%)	3 (3.3%)		1 (6.7%)	2 (2.6%)		2 (5.3%)	0 (0%)		3 (7.3%)	0 (0%)	
N	475 (94%)	381 (94%)	88 (96%)		14 (93%)	74 (96%)		36 (95%)	27 (100%)		37 (90%)	26 (96%)	
Missing	28	18	10		4	6		3	3		0	3	
**MCV**													
H	1 (0.2%)	0 (0%)	1 (1.1%)	0.20	0 (0%)	1 (1.3%)	1.00	1 (2.6%)	0 (0%)	0.51	2 (4.9%)	0 (0%)	0.30
L	10 (2.0%)	8 (2.0%)	2 (2.2%)		0 (0%)	2 (2.6%)		2 (5.3%)	0 (0%)		0 (0%)	1 (3.7%)	
N	495 (98%)	398 (98%)	89 (97%)		15 (100%)	74 (96%)		35 (92%)	27 (100%)		39 (95%)	26 (96%)	
Missing	28	18	10		4	6		3	3		0	3	
**MCH**													
H	4 (0.8%)	3 (0.7%)	1 (1.1%)	0.84	0 (0%)	1 (1.3%)	1.00	1 (2.6%)	0 (0%)	1.00	1 (2.4%)	0 (0%)	1.00
L	8 (1.6%)	7 (1.7%)	1 (1.1%)		0 (0%)	1 (1.3%)		1 (2.6%)	0 (0%)		1 (2.4%)	1 (3.7%)	
N	494 (98%)	396 (98%)	90 (98%)		15 (100%)	75 (97%)		36 (95%)	27 (100%)		39 (95%)	26 (96%)	
Missing	28	18	10		4	6		3	3		0	3	
**MCHC**													
H	105 (21%)	76 (19%)	26 (28%)	0.04	2 (13%)	24 (31%)	0.22	13 (34%)	4 (15%)	0.08	10 (24%)	2 (7.4%)	0.07
L	0 (0%)	0 (0%)	0(0%)		0 (0%)	0 (0%)		0 (0%)	0 (0%)		0 (0%)	0 (0%)	
N	401 (79%)	330 (81%)	66 (72%)		13 (87%)	53 (69%)		25 (66%)	23 (85%)		31 (76%)	25 (93%)	
Missing	28	18	10		4	6		3	3		0	3	

	6 months										12 months		
	COVID, N=534	No CMR abnormalities, N = 424	CMR abnormalities, N = 102	P	CMR abnormalities hospitalised, N = 19	CMR abnormalities non hospitalized, N = 83	P	Ongoing CMR abnormalities, N = 41	Resolved CMR abnormalities, N = 30	P	Ongoing CMR abnormalities, N = 41	Resolved CMR abnormalities, N = 30	P
**RDW**													
H	12 (2.4%)	10 (2.5%)	2 (2.2%)	0.94	0 (0%)	2 (2.6%)	1.00	2 (5.3%)	0 (0%)	0.53	0 (0%)	0 (0%)	1.00
L	26 (5.1%)	22 (5.4%)	4 (4.3%)		0 (0%)	4 (5.2%)		3 (7.9%)	1 (3.7%)		1 (2.4%)	0 (0%)	
N	467 (92%)	373 (92%)	86 (93%)		15 (100%)	71 (92%)		33 (87%)	26 (96%)		40 (98%)	27 (100%)	
Missing	29	19	10		4	6		3	3		0	3	
**Platelet count**													
H	22 (4.4%)	15 (3.7%)	6 (6.6%)	0.13	1 (6.7%)	5 (6.6%)	1.00	4 (11%)	1 (3.7%)	0.49	1 (2.4%)	3 (11%)	0.29
L	2 (0.4%)	1 (0.2%)	1 (1.1%)		0(0%)	1 (1.3%)		1 (2.7%)	0(0%)		0(0%)	0(0%)	
N	479 (95%)	388 (96%)	84 (92%)		14 (93%)	70 (92%)		32 (86%)	26 (96%)		40 (98%)	24 (89%)	
Missing	31	20	11		4	7		4	3		0	3	
**MPV**													
H	8 (1.6%)	6 (1.5%)	1 (1.1%)	1.00	0 (0%)	1 (1.3%)	1.00	1 (2.6%)	0 (0%)	1.00	2 (4.9%)	0 (0%)	0.51
L	0 (0%)	0 (0%)	0 (0%)		0 (0%)	0 (0%)		0(0%)	0 (0%)		0 (0%)	0 (0%)	
N	496 (98%)	398 (99%)	91 (99%)		15 (100%)	76 (99%)		37 (97%)	27 (100%)		39 (95%)	27 (100%)	
Missing	30	20	10		4	6		3	3		0	3	
**White cell count**													
H	19 (3.8%)	13 (3.2%)	3 (3.3%)	1.00	0 (0%)	3 (3.9%)	1.00	2 (5.3%)	0 (0%)	0.51	0 (0%)	0 (0%)	1.00
L	1 (0.2%)	1 (0.2%)	0 (0%)		0 (0%)	0 (0%)		0 (0%)	0 (0%)		0 (0%)	0 (0%)	
N	486 (96%)	392 (97%)	89 (97%)		15 (100%)	74 (96%)		36 (95%)	27 (100%)		41 (100%)	27 (100%)	
Missing	28	18	10		4	6		3	3		0	3	
**Neutrophils**													
H	8 (1.6%)	7 (1.7%)	1 (1.1%)	0.60	0 (0%)	1 (1.3%)	0.66	1 (2.6%)	0 (0%)	0.78	0 (0%)	0 (0%)	1.00
L	30 (5.9%)	20 (4.9%)	7 (7.6%)		0 (0%)	7 (9.1%)		3 (7.9%)	1 (3.7%)		3 (7.3%)	2 (7.4%)	
N	468 (92%)	379 (93%)	84 (91%)		15 (100%)	69 (90%)		34 (89%)	26 (96%)		38 (93%)	25 (93%)	
Missing	28	18	10		4	6		3	3		0	3	
**Lymphocytes**													
H	2 (0.4%)	1 (0.2%)	1 (1.1%)	0.27	0 (0%)	1 (1.3%)	0.65	0 (0%)	1 (3.7%)	0.59	1 (2.4%)	1 (3.7%)	0.22
L	38 (7.5%)	32 (7.9%)	5 (5.4%)		0 (0%)	5 (6.5%)		2 (5.3%)	2 (7.4%)		7 (17%)	1 (3.7%)	
N	466 (92%)	373 (92%)	86 (93%)		15 (100%)	71 (92%)		36 (95%)	24 (89%)		33 (80%)	25 (93%)	
Missing	28	18	10		4	6		3	3		0	3	
**Monocytes**													
H	4 (0.8%)	2 (0.5%)	1 (1.1%)	0.64	0 (0%)	1 (1.3%)	1.00	1 (2.6%)	0 (0%)	1.00	0 (0%)	0 (0%)	1.00
L	2 (0.4%)	2 (0.5%)	0 (0%)		0 (0%)	0 (0%)		0 (0%)	0 (0%)		0 (0%)	0 (0%)	
N	500 (99%)	402 (99%)	91 (99%)		15 (100%)	76 (99%)		37 (97%)	27 (100%)		41 (100%)	27 (100%)	
Missing	28	18	10		4	6		3	3		0	3	

Roca-Fernandez A, *et al. Open Heart* 2023; 10:e002241. doi: 10.1136/openhrt-2022-002241

	6 months										12 months		
	COVID, N=534	No CMR abnormalities, N = 424	CMR abnormalities, N = 102	P	CMR abnormalities hospitalised, N = 19	CMR abnormalities non hospitalized, N = 83	P	Ongoing CMR abnormalities, N = 41	Resolved CMR abnormalities, N = 30	P	Ongoing CMR abnormalities, N = 41	Resolved CMR abnormalities, N = 30	P
Eosinophils													
H<br>L<br>N<br>Missing	14 (2.8%)<br>0 (0%)<br>492 (97%)<br>28	10 (2.5%)<br>0 (0%)<br>396 (98%)<br>18	4 (4.3%)<br>0 (0%)<br>88 (96%)<br>10	0.30	0 (0%)<br>0 (0%)<br>15 (100%)<br>4	4 (5.2%)<br>0 (0%)<br>73 (95%)<br>6	1.00	0 (0%)<br>0 (0%)<br>38 (100%)<br>3	1 (3.7%)<br>0 (0%)<br>26 (96%)<br>3	0.42	2 (4.9%)<br>0 (0%)<br>39 (95%)<br>0	1 (3.7%)<br>0 (0%)<br>26 (96%)<br>3	1.00
Basophils													
H<br>L<br>N<br>Missing	2 (0.4%)<br>0 (0%)<br>504(100%)<br>28	2 (0.5%)<br>0(0%)<br>404 (100%)<br>18	0 (0%)<br>0 (0%)<br>92 (100%)<br>10	1.00	0 (0%)<br>0 (0%)<br>15 (100%)<br>4	0 (0%)<br>0 (0%)<br>77 (100%)<br>6	1.0	0 (0%)<br>0 (0%)<br>38 (100%)<br>3	0 (0%)<br>0 (0%)<br>27 (100%)<br>3	1.00	0 (0%)<br>0 (0%)<br>41 (100%)<br>0	2 (7.4%)<br>0 (0%)<br>25 (93%)<br>3	0.15
ESR													
H<br>L<br>N<br>Missing	40 (7.9%)<br>0 (0%)<br>468 (92%)<br>26	32 (7.9%)<br>0 (0%)<br>375 (92%)<br>17	8 (8.6%)<br>0 (0%)<br>85 (91%)<br>9	0.81	1 (6.7%)<br>0 (0%)<br>14 (93%)<br>4	7 (9.0%)<br>0 (0%)<br>71 (91%)<br>5	1.00	4 (11%)<br>0 (0%)<br>34 (89%)<br>3	3 (11%)<br>0 (0%)<br>24 (89%)<br>3	1.00	3 (7.3%)<br>0 (0%)<br>38 (93%)<br>0	4 (15%)<br>0 (0%)<br>23 (85%)<br>3	1.0
Sodium													
H<br>L<br>N<br>Missing	1 (0.2%)<br>17 (3.4%)<br>488 (96%)<br>28	1 (0.2%)<br>13 (3.2%)<br>392 (97%)<br>18	0 (0%)<br>4 (4.3%)<br>88 (96%)<br>10	0.62	0 (0%)<br>0 (0%)<br>15 (100%)<br>4	0 (0%)<br>4 (5.2%)<br>73 (95%)<br>6	1.00	0 (0%)<br>4 (11%)<br>34 (89%)<br>3	0 (0%)<br>0 (0%)<br>27 (100%)<br>3	0.13	0 (0%)<br>4 (9.8%)<br>37 (90%)<br>0	0 (0%)<br>0 (0%)<br>28 (100%)<br>2	0.14
Potassium													
H<br>L<br>N<br>Missing	231 (49%)<br>0 (0%)<br>243 (51%)<br>60	188 (49%)<br>0 (0%)<br>194 (51%)<br>42	42 (49%)<br>0 (0%)<br>43 (51%)<br>17	0.97	6 (46%)<br>0 (0%)<br>7 (54%)<br>6	36 (50%)<br>0 (0%)<br>36 (50%)<br>11	0.80	17 (47%)<br>0 (0%)<br>19 (53%)<br>5	12 (52%)<br>0 (0%)<br>11 (48%)<br>7	0.71	8 (32%)<br>0 (0%)<br>17 (68%)<br>16	9 (47%)<br>0 (0%)<br>10 (53%)<br>11	0.30
Chloride													
H<br>L<br>N<br>Missing	10 (2.0%)<br>11 (2.2%)<br>485 (96%)<br>28	7 (1.7%)<br>6 (1.5%)<br>393 (97%)<br>18	2 (2.2%)<br>5 (5.4%)<br>85 (92%)<br>10	0.05	0 (0%)<br>0 (0%)<br>15 (100%)<br>4	2 (2.6%)<br>5 (6.5%)<br>70 (91%)<br>6	0.71	1 (2.6%)<br>3 (7.9%)<br>34 (89%)<br>3	0 (0%)<br>0 (0%)<br>27 (100%)<br>3	0.26	0 (0%)<br>2 (4.9%)<br>39 (95%)<br>0	0 (0%)<br>0 (0%)<br>28 (100%)<br>2	0.51
Bicarbonate													
H<br>L<br>N<br>Missing	25 (4.9%)<br>49 (9.7%)<br>432 (85%)<br>28	18 (4.4%)<br>37 (9.1%)<br>351 (86%)<br>18	7 (7.6%)<br>10 (11%)<br>75 (82%)<br>10	0.30	2 (13%)<br>0 (0%)<br>13 (87%)<br>4	5 (6.5%)<br>10 (13%)<br>62 (81%)<br>6	0.24	4 (11%)<br>4 (11%)<br>30 (79%)<br>3	2 (7.4%)<br>4 (15%)<br>21 (78%)<br>3	0.82	2 (4.9%)<br>1 (2.4%)<br>38 (93%)<br>0	1 (3.6%)<br>1 (3.6%)<br>26 (93%)<br>2	1.00
Urea													

	6 months										12 months		
	COVID, N=534	No CMR abnormalities, N = 424	CMR abnormalities, N = 102	P	CMR abnormalities hospitalised, N = 19	CMR abnormalities non hospitalized, N = 83	P	Ongoing CMR abnormalities, N = 41	Resolved CMR abnormalities, N = 30	P	Ongoing CMR abnormalities, N = 41	Resolved CMR abnormalities, N = 30	P
H<br>L<br>N<br>Missing	1 (0.2%)<br>1 (0.2%)<br>504(100%)<br>28	0 (0%)<br>1 (0.2%)<br>405 (100%)<br>18	0 (0%)<br>0 (0%)<br>92 (100%)<br>10	1.00	0 (0%)<br>0 (0%)<br>15 (100%)<br>4	0 (0%)<br>0 (0%)<br>77 (100%)<br>6	1.00	0 (0%)<br>0 (0%)<br>38 (100%)<br>3	0 (0%)<br>0 (0%)<br>27 (100%)<br>3	1.00	1 (2.4%)<br>0 (0%)<br>40 (98%)<br>0	0 (0%)<br>0 (0%)<br>28 (100%)<br>2	1.00
Creatinine													
H<br>L<br>N<br>Missing	6 (1.2%)<br>25 (4.9%)<br>475 (94%)<br>28	6 (1.5%)<br>20 (4.9%)<br>380 (94%)<br>18	0 (0%)<br>5 (5.4%)<br>87 (95%)<br>10	0.65	0 (0%)<br>2 (13%)<br>13 (87%)<br>4	0 (0%)<br>3 (3.9%)<br>74 (96%)<br>6	0.19	0 (0%)<br>2 (5.3%)<br>36 (95%)<br>3	0 (0%)<br>2 (7.4%)<br>25 (93%)<br>3	1.00	1 (2.4%)<br>2 (4.9%)<br>38 (93%)<br>0	0 (0%)<br>2 (7.1%)<br>26 (93%)<br>2	1.00
Bilirubin													
H<br>L<br>N<br>Missing	17 (3.4%)<br>0 (0%)<br>489 (97%)<br>28	11 (2.7%)<br>0 (0%)<br>395 (97%)<br>18	4 (4.3%)<br>0 (0%)<br>88 (96%)<br>10	0.49	1 (6.7%)<br>0 (0%)<br>14 (93%)<br>4	3 (3.9%)<br>0 (0%)<br>74 (96%)<br>6	0.52	3 (7.9%)<br>0 (0%)<br>35 (92%)<br>3	0 (0%)<br>0 (0%)<br>27 (100%)<br>3	0.26	2 (4.9%)<br>0 (0%)<br>39 (95%)<br>0	2 (7.1%)<br>0 (0%)<br>26 (93%)<br>2	1.00
Alkaline phosphatase													
H<br>L<br>N<br>Missing	12 (2.4%)<br>13 (2.6%)<br>481 (95%)<br>28	9 (2.2%)<br>9 (2.2%)<br>388 (96%)<br>18	2 (2.2%)<br>4 (4.3%)<br>86 (93%)<br>10	0.44	0 (0%)<br>0 (0%)<br>15 (100%)<br>4	2 (2.6%)<br>4 (5.2%)<br>71 (92%)<br>6	1.00	1 (2.6%)<br>2 (5.3%)<br>35 (92%)<br>3	0 (0%)<br>1 (3.7%)<br>26 (96%)<br>3	1.00	0 (0%)<br>0 (0%)<br>41 (100%)<br>0	0 (0%)<br>1 (3.6%)<br>27 (96%)<br>2	0.41
Aspartate transferase													
H<br>L<br>N<br>Missing	43 (8.8%)<br>0 (0%)<br>443 (91%)<br>48	33 (8.4%)<br>0 (0%)<br>359 (92%)<br>32	10 (12%)<br>0 (0%)<br>76 (88%)<br>16	0.35	1 (7.1%)<br>0 (0%)<br>13 (93%)<br>5	9 (12%)<br>0 (0%)<br>63 (88%)<br>11	1.00	4 (11%)<br>0 (0%)<br>31 (89%)<br>6	2 (8.3%)<br>0 (0%)<br>22 (92%)<br>6	1.00	6 (15%)<br>0 (0%)<br>34 (85%)<br>1	2 (8.0%)<br>0 (0%)<br>23 (92%)<br>5	0.47
Alanine transferase													
H<br>L<br>N<br>Missing	73 (14%)<br>7 (1.4%)<br>426 (84%)<br>28	62 (15%)<br>6 (1.5%)<br>338 (83%)<br>18	11 (12%)<br>1 (1.1%)<br>80 (87%)<br>10	0.81	1 (6.7%)<br>0 (0%)<br>14 (93%)<br>4	10 (13%)<br>1 (1.3%)<br>66 (86%)<br>6	0.74	5 (13%)<br>0 (0%)<br>33 (87%)<br>3	4 (15%)<br>1 (3.7%)<br>22 (81%)<br>3	0.69	7 (17%)<br>1 (2.4%)<br>33 (80%)<br>0	5 (18%)<br>0 (0%)<br>23 (82%)<br>2	1.00
LDH													
H<br>L<br>N<br>Missing	80 (16%)<br>18 (3.6%)<br>400 (80%)<br>36	63 (16%)<br>12 (3.0%)<br>326 (81%)<br>23	16 (18%)<br>3 (3.4%)<br>70 (79%)<br>13	0.79	1 (6.7%)<br>0 (0%)<br>14 (93%)<br>4	15 (20%)<br>3 (4.1%)<br>56 (76%)<br>9	0.40	9 (25%)<br>0 (0%)<br>27 (75%)<br>5	4 (15%)<br>2 (7.7%)<br>20 (77%)<br>4	0.19	10 (24%)<br>0 (0%)<br>31 (76%)<br>0	7 (26%)<br>0 (0%)<br>20 (74%)<br>3	0.89
CK													
H<br>L	40 (7.9%)<br>2 (0.4%)	31 (7.6%)<br>1 (0.2%)	9 (9.8%)<br>1 (1.1%)	0.28	1 (6.7%)<br>0 (0%)	8 (10%)<br>1 (1.3%)	1.00	3 (7.9%)<br>1 (2.6%)	3 (11%)<br>0 (0%)	0.82	4 (9.8%)<br>1 (2.4%)	4 (15%)<br>0 (0%)	0.82

	6 months										12 months		
	COVID, N=534	No CMR abnormalities, N = 424	CMR abnormalities, N = 102	P	CMR abnormalities hospitalised, N = 19	CMR abnormalities non hospitalized, N = 83	P	Ongoing CMR abnormalities, N = 41	Resolved CMR abnormalities, N = 30	P	Ongoing CMR abnormalities, N = 41	Resolved CMR abnormalities, N = 30	P
N<br>Missing	464 (92%)<br>28	374 (92%)<br>18	82 (89%)<br>10		14 (93%)<br>4	68 (88%)<br>6		34 (89%)<br>3	24 (89%)<br>3		36 (88%)<br>0	23 (85%)<br>3	
Gamma GT													
H<br>L<br>N<br>Missing	32 (6.3%)<br>12 (2.4%)<br>462 (91%)<br>28	26 (6.4%)<br>8 (2.0%)<br>372 (92%)<br>18	5 (5.4%)<br>3 (3.3%)<br>84 (91%)<br>10	0.66	0 (0%)<br>0 (0%)<br>15 (100%)<br>4	5 (6.5%)<br>3 (3.9%)<br>69 (90%)<br>6	0.76	3 (7.9%)<br>2 (5.3%)<br>33 (87%)<br>3	1 (3.7%)<br>0 (0%)<br>26 (96%)<br>3	0.53	3 (7.3%)<br>3 (7.3%)<br>35 (85%)<br>0	0 (0%)<br>0 (0%)<br>28 (100%)<br>2	0.16
Total protein													
H<br>L<br>N<br>Missing	2 (0.4%)<br>7 (1.4%)<br>497 (98%)<br>28	1 (0.2%)<br>5 (1.2%)<br>400 (99%)<br>18	1 (1.1%)<br>2 (2.2%)<br>89 (97%)<br>10	0.30	0 (0%)<br>0 (0%)<br>15 (100%)<br>4	1 (1.3%)<br>2 (2.6%)<br>74 (96%)<br>6	1.00	1 (2.6%)<br>1 (2.6%)<br>36 (95%)<br>3	0 (0%)<br>1 (3.7%)<br>26 (96%)<br>3	1.00	0 (0%)<br>1 (2.4%)<br>40 (98%)<br>0	0 (0%)<br>0 (0%)<br>28 (100%)<br>2	1.00
Albumin													
H<br>L<br>N<br>Missing	27 (5.3%)<br>0 (0%)<br>479 (95%)<br>28	21 (5.2%)<br>0 (0%)<br>385 (95%)<br>18	6 (6.5%)<br>0 (0%)<br>86 (93%)<br>10	0.61	0 (0%)<br>0 (0%)<br>15 (100%)<br>4	6 (7.8%)<br>0 (0%)<br>71 (92%)<br>6	0.58	4 (11%)<br>0 (0%)<br>34 (89%)<br>3	2 (7.4%)<br>0 (0%)<br>25 (93%)<br>3	1.00	1 (2.4%)<br>0 (0%)<br>40 (98%)<br>0	0 (0%)<br>0 (0%)<br>28 (100%)<br>2	1.00
Globulin													
H<br>L<br>N<br>Missing	2 (0.4%)<br>14 (2.8%)<br>490 (97%)<br>28	2 (0.5%)<br>11 (2.7%)<br>393 (97%)<br>18	0 (0%)<br>3 (3.3%)<br>89 (97%)<br>10	0.82	0 (0%)<br>0 (0%)<br>15 (100%)<br>4	0 (0%)<br>3 (3.9%)<br>74 (96%)<br>6	1.00	0 (0%)<br>2 (5.3%)<br>36 (95%)<br>3	0 (0%)<br>0 (0%)<br>27 (100%)<br>3	0.51	0 (0%)<br>1 (2.4%)<br>40 (98%)<br>0	0 (0%)<br>0 (0%)<br>28 (100%)<br>2	1.00
Calcium													
H<br>L<br>N<br>Missing	7 (1.4%)<br>8 (1.6%)<br>491 (97%)<br>28	4 (1.0%)<br>6 (1.5%)<br>396 (98%)<br>18	3 (3.3%)<br>2 (2.2%)<br>87 (95%)<br>10	0.12	0 (0%)<br>0 (0%)<br>15 (100%)<br>4	3 (3.9%)<br>2 (2.6%)<br>72 (94%)<br>6	1.00	2 (5.3%)<br>0 (0%)<br>36 (95%)<br>3	1 (3.7%)<br>1 (3.7%)<br>25 (93%)<br>3	0.75	0 (0%)<br>1 (2.4%)<br>40 (98%)<br>0	0 (0%)<br>0 (0%)<br>28 (100%)<br>2	1.00
Magnesium													
H<br>L<br>N<br>Missing	2 (0.4%)<br>1 (0.2%)<br>503 (99%)<br>28	2 (0.5%)<br>0 (0%)<br>404 (100%)<br>18	0 (0%)<br>1 (1.1%)<br>91 (99%)<br>10	0.21	0 (0%)<br>0 (0%)<br>15 (100%)<br>4	0 (0%)<br>1 (1.3%)<br>76 (99%)<br>6	1.00	0 (0%)<br>1 (2.6%)<br>37 (97%)<br>3	0 (0%)<br>0 (0%)<br>27 (100%)<br>3	1.00	0 (0%)<br>1 (2.4%)<br>40 (98%)<br>0	0 (0%)<br>0 (0%)<br>28 (100%)<br>2	1.00
Phosphate													
H<br>L<br>N<br>Missing	13 (2.6%)<br>53 (10%)<br>440 (87%)<br>28	8 (2.0%)<br>46 (11%)<br>352 (87%)<br>18	5 (5.4%)<br>6 (6.5%)<br>81 (88%)<br>10	0.08	1 (6.7%)<br>0 (0%)<br>14 (93%)<br>4	4 (5.2%)<br>6 (7.8%)<br>67 (87%)<br>6	0.66	2 (5.3%)<br>3 (7.9%)<br>33 (87%)<br>3	2 (7.4%)<br>3 (11%)<br>22 (81%)<br>3	0.88	2 (4.9%)<br>4 (9.8%)<br>35 (85%)<br>0	2 (7.1%)<br>5 (18%)<br>21 (75%)<br>2	0.56

	6 months										12 months		
	COVID, N=534	No CMR abnormalities, N = 424	CMR abnormalities, N = 102	P	CMR abnormalities hospitalised, N = 19	CMR abnormalities non hospitalized, N = 83	P	Ongoing CMR abnormalities, N = 41	Resolved CMR abnormalities, N = 30	P	Ongoing CMR abnormalities, N = 41	Resolved CMR abnormalities, N = 30	P
**Uric acid**													
H	29 (5.7%)	22 (5.4%)	7 (7.6%)	0.71	3 (20%)	4 (5.2%)	0.03	1 (2.6%)	4 (15%)	0.21	1 (2.4%)	4 (14%)	0.11
L	59 (12%)	48 (12%)	10 (11%)		3 (20%)	7 (9.1%)		4 (11%)	2 (7.4%)		7 (17%)	2 (7.1%)	
N	418 (83%)	336 (83%)	75 (82%)		9 (60%)	66 (86%)		33 (87%)	21 (78%)		33 (80%)	22 (79%)	
Missing	28	18	10		4	6		3	3		0	2	
**Triglycerides**													
H	26 (20%)	20 (20%)	4 (17%)	1.00	0 (0%)	4 (19%)	1.00	3 (27%)	1 (20%)	1.00	3 (10%)	5 (26%)	0.24
L	0 (0%)	0 (0%)	0 (0%)		0(0%)	0 (0%)		0 (0%)	0 (0%)		0 (0%)	0 (0%)	
N	102 (80%)	81 (80%)	20 (83%)		3 (100%)	17 (81%)		8 (73%)	4 (80%)		26 (90%)	14 (74%)	
Missing	406	323	78		16	62		30	25		12	11	
**Fasting triglycerides**													
H	44 (12%)	39 (13%)	5 (7.4%)	0.21	1 (8.3%)	4 (7.1%)	1.00	2 (7.4%)	1 (4.5%)	1.00	1 (8.3%)	0 (0%)	1.00
L	0 (0%)	0 (0%)	0 (0%)		0 (0%)	0 (0%)		0 (0%)	0 (0%)		0 (0%)	0 (0%)	
N	334 (88%)	266 (87%)	63 (93%)		11 (92%)	52 (93%)		25 (93%)	21 (95%)		11 (92%)	9 (100%)	
Missing	156	119	34		7	27		14	8		29	21	
**Cholesterol**													
H	68 (53%)	54 (53%)	13 (54%)	0.95	1 (33%)	12 (57%)	0.58	5 (45%)	3 (60%)	1.00	10 (34%)	8 (42%)	0.86
L	0 (0%)	0 (0%)	0 (0%)		0 (0%)	0 (0%)		0 (0%)	0 (0%)		1 (3.4%)	0 (0%)	
N	60 (47%)	47 (47%)	11 (46%)		2 (67%)	9 (43%)		6 (55%)	2 (40%)		18 (62%)	11 (58%)	
Missing	406	323	78		16	62		30	25		12	11	
**Fasting cholesterol**													
H	167 (44%)	143 (47%)	24 (35%)	0.08	4 (33%)	20 (36%)	1.00	8 (30%)	8 (36%)	0.62	9 (75%)	5 (56%)	0.40
L	0 (0%)	0 (0%)	0 (0%)		0 (0%)	0 (0%)		0 (0%)	0 (0%)		0 (0%)	0 (0%)	
N	211 (56%)	162 (53%)	44 (65%)		8 (67%)	36 (64%)		19 (70%)	14 (64%)		3 (25%)	4 (44%)	
Missing	156	119	34		7	27		14	8		29	21	
**HDL cholesterol**													
H	176 (35%)	141 (35%)	31 (34%)	0.94	6 (40%)	25 (32%)	0.91	13 (34%)	7 (26%)	0.72	12 (29%)	6 (21%)	0.22
L	40 (7.9%)	31 (7.6%)	8 (8.7%)		1 (6.7%)	7 (9.1%)		5 (13%)	3 (11%)		2 (4.9%)	5 (18%)	
N	290 (57%)	234 (58%)	53 (58%)		8 (53%)	45 (58%)		20 (53%)	17 (63%)		27 (66%)	17 (61%)	
Missing	28	18	10		4	6		3	3		0	2	
**LDL cholesterol**													
H	166 (33%)	137 (34%)	28 (31%)	0.63	5 (36%)	23 (31%)	0.71	8 (22%)	9 (35%)	0.28	15 (37%)	9 (32%)	0.70
L	0 (0%)	0 (0%)	0 (0%)		0 (0%)	0 (0%)		0 (0%)	0 (0%)		0 (0%)	0 (0%)	
N	332 (67%)	264 (66%)	61 (69%)		9 (64%)	52 (69%)		28 (78%)	17 (65%)		26 (63%)	19 (68%)	
Missing	36	23	13		5	8		5	4		0	2	
**Iron**													

	6 months										12 months		
	COVID, N=534	No CMR abnormalities, N = 424	CMR abnormalities, N = 102	P	CMR abnormalities hospitalised, N = 19	CMR abnormalities non hospitalized, N = 83	P	Ongoing CMR abnormalities, N = 41	Resolved CMR abnormalities, N = 30	P	Ongoing CMR abnormalities, N = 41	Resolved CMR abnormalities, N = 30	P
H	24 (4.7%)	19 (4.7%)	4 (4.3%)	1.00	0 (0%)	4 (5.2%)	1.00	3 (7.9%)	1 (3.7%)	0.78	1 (2.4%)	0 (0%)	1.00
L	10 (2.0%)	8 (2.0%)	2 (2.2%)		0 (0%)	2 (2.6%)		1 (2.6%)	0 (0%)		2 (4.9%)	2 (7.1%)	
N	472 (93%)	379 (93%)	86 (93%)		15 (100%)	71 (92%)		34 (89%)	26 (96%)		38 (93%)	26 (93%)	
Missing	28	18	10		4	6		3	3		0	2	
TIBC													
H	19 (3.8%)	15 (3.7%)	3 (3.3%)	1.00	0 (0%)	3 (4.0%)	1.00	2 (5.4%)	0 (0%)	0.51	1 (2.4%)	0 (0%)	0.64
L	1 (0.2%)	1 (0.2%)	0 (0%)		0 (0%)	0 (0%)		0 (0%)	0 (0%)		0 (0%)	1 (3.7%)	
N	479 (96%)	385 (96%)	87 (97%)		15 (100%)	72 (96%)		35 (95%)	26 (100%)		40 (98%)	26 (96%)	
Missing	35	23	12		4	8		4	4		0	3	
Transferrin saturation													
H	9 (1.8%)	8 (2.0%)	1 (1.1%)	0.64	0 (0%)	1 (1.3%)	1.00	1 (2.7%)	0 (0%)	0.86	0 (0%)	0 (0%)	1.00
L	78 (16%)	60 (15%)	17 (19%)		3 (20%)	14 (19%)		7 (19%)	6 (23%)		6 (15%)	3 (11%)	
N	412 (83%)	333 (83%)	72 (80%)		12 (80%)	60 (80%)		29 (78%)	20 (77%)		35 (85%)	24 (89%)	
Missing	35	23	12		4	8		4	4		0	3	
CRP highly sensitive													
H	37 (7.3%)	33 (8.1%)	4 (4.3%)	0.21	1 (6.7%)	3 (3.9%)	0.52	0 (0%)	2 (7.4%)	0.17	2 (4.9%)	2 (7.1%)	1.00
L	0 (0%)	0 (0%)	0 (0%)		0 (0%)	0 (0%)		0(0%)	0 (0%)		0 (0%)	0 (0%)	
N	468 (93%)	372 (92%)	88 (96%)		14 (93%)	74 (96%)		38 (100%)	25 (93%)		39 (95%)	26 (93%)	
Missing	29	19	10		4	6		3	3		0	2	
Troponin I highly sensitive													
H	4 (0.9%)	4 (1.1%)	0 (0%)	1.00	0 (0%)	0 (0%)	1.00	0 (0%)	0 (0%)	1.00	0 (0%)	0 (0%)	1.00
L	0 (0%)	0 (0%)	0 (0%)		0 (0%)	0 (0%)		0 (0%)	0 (0%)		0 (0%)	0 (0%)	
N	458(99%)	368 (99%)	83 (100%)		18 (100%)	65 (100%)		33 (100%)	24 (100%)		32 (100%)	24 (100%)	
Missing	72	52	19		1	18		8	6		9	6	
Amylase													
H	34 (7.3%)	23 (6.2%)	8 (9.6%)	0.03	2 (11%)	6 (9.2%)	0.73	3 (9.1%)	3 (12%)	0.84	3 (9.4%)	4 (17%)	0.82
L	10 (2.2%)	5 (1.3%)	5 (6.0%)		0 (0%)	5 (7.7%)		1 (3.0%)	1 (4.2%)		1 (3.1%)	0 (0%)	
N	418 (91%)	344 (92%)	70 (84%)		16 (89%)	54 (83%)		29 (88%)	20 (83%)		28 (88%)	20 (83%)	
Missing	72	52	19		1	18		8	6		9	6	
Ferritin													
H	61 (13.2%)	48 (13%)	13 (16%)	0.76	2 (11%)	11 (17%)	0.78	6 (18%)	3 (12%)	0.72	6 (19%)	4 (17%)	1.00
L	11 (2.4%)	10 (2.7%)	1 (1.2%)		0 (0%)	1 (1.5%)		0 (0%)	0 (0%)		1 (3.1%)	0 (0%)	
N	390 (84%)	314 (84%)	69 (83%)		16 (89%)	53 (82%)		27 (82%)	21 (88%)		25 (78%)	20 (83%)	
Missing	72	52	19		1	18		8	6		9	6	
Lipase													
H	36 (7.7%)	29 (7.7%)	5 (5.9%)	0.88	1 (5.6%)	4 (6.0%)	0.26	2 (5.9%)	2 (8.0%)	1.00	3 (9.4%)	1 (4.2%)	0.63

	6 months										12 months		
	COVID, N=534	No CMR abnormalities, N = 424	CMR abnormalities, N = 102	P	CMR abnormalities hospitalised, N = 19	CMR abnormalities non hospitalized, N = 83	P	Ongoing CMR abnormalities, N = 41	Resolved CMR abnormalities, N = 30	P	Ongoing CMR abnormalities, N = 41	Resolved CMR abnormalities, N = 30	P
L<br>N<br>Missing	5 (1.1%)<br>425 (91%)<br>68	4 (1.1%)<br>341 (91%)<br>50	1 (1.2%)<br>79 (93%)<br>17		1 (5.6%)<br>16 (89%)<br>1	0 (0%)<br>63 (94%)<br>16		0 (0%)<br>32 (94%)<br>7	0 (0%)<br>23 (92%)<br>5		0 (0%)<br>29 (91%)<br>9	0 (0%)<br>23 (96%)<br>6	
Thyroid stimulating hormone													
H<br>L<br>N<br>Missing	3 (0.6%)<br>0 (0%)<br>464 (99%)<br>67	3 (0.8%)<br>0 (0%)<br>372 (99%)<br>49	0 (0%)<br>0 (0%)<br>85 (100%)<br>17	1.00	0 (0%)<br>0 (0%)<br>18 (100%)<br>1	0 (0%)<br>0 (0%)<br>67 (100%)<br>16	1.00	0 (0%)<br>0 (0%)<br>34 (100%)<br>7	0 (0%)<br>0 (0%)<br>25 (100%)<br>5	1.00	1 (3.1%)<br>0 (0%)<br>31 (97%)<br>9	1 (4.2%)<br>0 (0%)<br>23 (96%)<br>6	1.00
Testosterone													
H<br>L<br>N<br>Missing	19 (4.1%)<br>9 (1.9%)<br>434 (94%)<br>72	17 (4.6%)<br>6 (1.6%)<br>349 (94%)<br>52	1 (1.2%)<br>3 (3.6%)<br>79 (95%)<br>19	0.30	0 (0%)<br>3 (17%)<br>15 (83%)<br>1	1 (1.5%)<br>0 (0%)<br>64 (98%)<br>18	0.01	0 (0%)<br>1 (3.0%)<br>32 (97%)<br>8	0 (0%)<br>1 (4.2%)<br>23 (96%)<br>6	1.00	0 (0%)<br>2 (6.2%)<br>30 (94%)<br>9	0 (0%)<br>2 (8.3%)<br>22 (92%)<br>6	1.00
Insulin													
H<br>L<br>N<br>Missing	41 (8.9%)<br>10 (2.2%)<br>408 (89%)<br>75	32 (8.6%)<br>7 (1.9%)<br>332 (89%)<br>53	8 (9.9%)<br>3 (3.7%)<br>70 (86%)<br>21	0.59	2 (12%)<br>0 (0%)<br>15 (88%)<br>2	6 (9.4%)<br>3 (4.7%)<br>55 (86%)<br>19	1.00	3 (9.4%)<br>1 (3.1%)<br>28 (88%)<br>9	4 (17%)<br>0 (0%)<br>19 (83%)<br>7	0.68	3 (9.4%)<br>0 (0%)<br>29 (91%)<br>9	3 (12%)<br>0 (0%)<br>21 (88%)<br>6	1.00
C peptide													
H<br>L<br>N<br>Missing	19 (4.1%)<br>0 (0%)<br>443 (96%)<br>72	16 (4.3%)<br>0 (0%)<br>357 (96%)<br>51	2 (2.4%)<br>0 (0%)<br>80 (98%)<br>20	0.75	1 (5.9%)<br>0 (0%)<br>16 (94%)<br>2	1 (1.5%)<br>0 (0%)<br>64 (98%)<br>18	0.37	2 (6.1%)<br>0 (0%)<br>31 (94%)<br>8	0 (0%)<br>0 (0%)<br>23 (100%)<br>7	0.51	3 (9.4%)<br>0 (0%)<br>29 (91%)<br>9	3 (12%)<br>0 (0%)<br>21 (88%)<br>6	1.00
NT-proBNP													
H<br>L<br>N<br>Missing	2 (0.4%)<br>0 (0%)<br>460 (99%)<br>72	1 (0.3%)<br>0 (0%)<br>371 (99%)<br>52	1 (1.2%)<br>0 (0%)<br>82 (99%)<br>19	0.45	1 (5.6%)<br>0 (0%)<br>17 (94%)<br>1	0 (0%)<br>0 (0%)<br>65 (100%)<br>18	0.22	0 (0%)<br>0 (0%)<br>33 (100%)<br>8	1 (4.2%)<br>0 (0%)<br>23 (96%)<br>6	0.42	0 (0%)<br>0 (0%)<br>32 (100%)<br>9	0 (0%)<br>0 (0%)<br>24 (100%)<br>6	1.00

Values presented as count and %. Cells with red shading indicating significant differences

Abbreviations: H, high; L, Low; N, Normal range; HCT, haematocrit test; MCV, Mean corpuscular volume; MCH, mean corpuscular haemoglobin; MCHC, mean corpuscular haemoglobin concentration; RDW, red cell distribution width; MPV, mean platelet volume; ESR, erythrocyte sedimentation rate; LDH, Lactate dehydrogenase; CK, Creatine Kinase; HDL, high-density lipoprotein; LDL, low-density lipoprotein; TIBC, total iron-binding capacity; CRP, C-reactive protein; NT-proBNP, N-terminal pro B-type natriuretic peptide.

Roca-Fernandez A, *et al. Open Heart* 2023; 10:e002241. doi: 10.1136/openhrt-2022-002241

Figure S5: T1 topographical abnormalities in participants classified as CMR abnormal at 6 months (n=102)

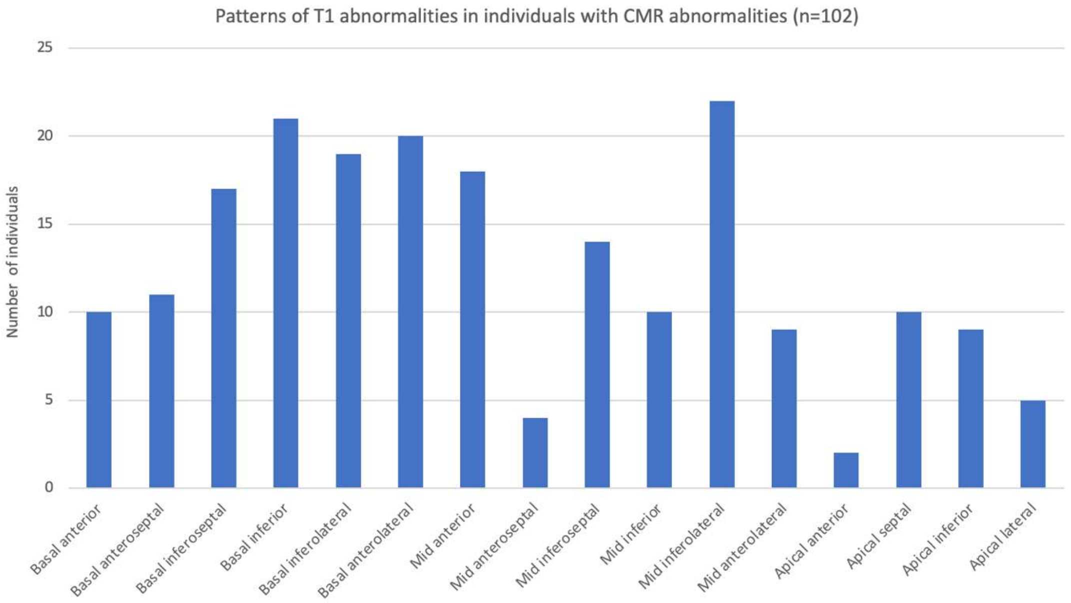

Table S6. Detailed CMR findings in new onset cardiac abnormalities at 12 months. Red cells indicate abnormal values

		A	B	C	D	E	F	G	H	I	J
Field Strength		1.5T	1.5T	1.5T	1.5T	1.5T	3T	1.5T	1.5T	3T	3T
sex		F	F	F	M	M	F	F	M	M	F
Age range		56-60	41-45	46-50	51-55	66-70	46-50	46-50	56-60	30-35	30-35
Global T1	baseline	1018	968	976	985	952	1219	1001	942	1141	1182
	follow up	1025	1018	1024	998	988	1278	998	934	1170	1238
≥ 3 elevated T1 segments	baseline	No	No	No	No	No	No	No	No	No	No
	follow up	Yes	Yes	Yes	Yes	Yes	Yes	No	No	No	Yes
Global T2	follow up	48	NA	47	47	47	45	NA	46	NA	NA
≥ 3 elevated T2 segments	follow up	No	No	No	No	No	Yes	No	No	No	No
Left end diastolic volume (mL)	baseline	65	81	80	89	81	90	75	117	80	73
	follow up	64	91	83	78	86	91	68	103	79	54
Left end systolic volume (mL)	baseline	27	29	34	35	28	34	29	49	34	28
	follow up	23	31	31	36	33	38	30	49	35	27
Left ejection fraction (%)	baseline	58	64	58	61	65	63	62	58	57	62
	follow up	64	66	63	55	61	58	56	53	55	51
Left stroke volume (mL)	baseline	38	52	46	54	53	57	47	68	46	45
	follow up	41	60	52	42	53	53	38	54	44	27
Left ventricular max wall thickness (mm)	baseline	10	9	7	9	9	9	8	10	10	9
	follow up	11	8	8	12	9	7	9	12	8	11
Left ventricular muscle mass (mm)	baseline	79	79	61	99	84	56	63	134	74	83
	follow up	89	75	65	118	82	58	67	149	70	92
Left global circumferential strain (%)	baseline	-20	-25	-24	-23	-21	-20	-24	-19	-20	-23
	follow up	-21	-25	-24	-19	-20	-22	NA	-17	-19	-19
Left global longitudinal strain (%)	baseline	-13	-18	-14	-15	-15	-15	-16	-9	-14	-16
	follow up	-13	-17	-18	-16	-12	-17	NA	-10	-13	-14
Right end diastolic volume (mL)	baseline	68	79	80	86	94	90	77	132	75	77
	follow up	62	81	78	56	98	92	71	130	86	56
Right end systolic volume (mL)	baseline	31	28	34	32	39	40	31	60	33	28
	follow up	31	28	31	23	41	41	37	71	44	28
Right ejection fraction (%)	baseline	54	64	58	63	58	56	59	55	56	63
	follow up	50	65	60	58	58	55	48	45	49	49
Right stroke volume (mL)	baseline	36	51	46	54	55	50	46	72	42	48
	follow up	31	52	47	33	57	51	34	59	42	27

Roca-Fernandez A, *et al. Open Heart* 2023; 10:e002241. doi: 10.1136/openhrt-2022-002241

Supplementary References:

1. Yorke J, Moosavi SH, Shuldham C, Jones PW. Quantification of dyspnoea using descriptors: development and initial testing of the Dyspnoea-12. Thorax. 2010 Jan;65(1):21–6.

2. Hobbins A, Barry L, Kelleher D, O'Neill C. The health of the residents of Ireland: Population norms for Ireland based on the EQ-5D-5L descriptive system - a cross sectional study. HRB Open Res. 2018;1:22.

3. Dennis A, Wamil M, Alberts J, Oben J, Cuthbertson DJ, Wootton D, et al. Multiorgan impairment in low-risk individuals with post-COVID-19 syndrome: a prospective, community-based study. BMJ Open. 2021 Mar 1;11(3):e048391.

4. Janssen MF, Pickard AS, Golicki D, Gudex C, Niewada M, Scalone L, et al. Measurement properties of the EQ-5D-5L compared to the EQ-5D-3L across eight patient groups: a multi-country study. Qual Life Res. 2013 Sep;22(7):1717–27.

5. Petersen SE, Matthews PM, Francis JM, Robson MD, Zemrak F, Boubertakh R, et al. UK Biobank's cardiovascular magnetic resonance protocol. J Cardiovasc Magn Reson. 2016 Feb 1;18:8.

6. Ferreira VM, Piechnik SK, Dall'Armellina E, Karamitsos TD, Francis JM, Ntusi N, et al. Native T1-mapping detects the location, extent and patterns of acute myocarditis without the need for gadolinium contrast agents. Journal of Cardiovascular Magnetic Resonance. 2014 May 23;16(1):36.

7. Ferreira VM, Schulz-Menger J, Holmvang G, Kramer CM, Carbone I, Sechtem U, et al. Cardiovascular Magnetic Resonance in Nonischemic Myocardial Inflammation: Expert Recommendations. J Am Coll Cardiol. 2018 Dec 18;72(24):3158–76.

8. Pavlides M, Banerjee R, Tunnicliffe EM, Kelly C, Collier J, Wang LM, et al. Multiparametric magnetic resonance imaging for the assessment of non-alcoholic fatty liver disease severity. Liver Int. 2017;37(7):1065–73.

9. Petersen SE, Aung N, Sanghvi MM, Zemrak F, Fung K, Paiva JM, et al. Reference ranges for cardiac structure and function using cardiovascular magnetic resonance (CMR) in Caucasians from the UK Biobank population cohort. J Cardiovasc Magn Reson. 2017 Feb 3;19(1):18.

10. Cerqueira MD, Weissman NJ, Dilsizian V, Jacobs AK, Kaul S, Laskey WK, et al. Standardized myocardial segmentation and nomenclature for tomographic imaging of the heart. A statement for healthcare professionals from the Cardiac Imaging Committee of the Council on Clinical Cardiology of the American Heart Association. J Nucl Cardiol. 2002 Apr;9(2):240–5.

11. Sudlow C, Gallacher J, Allen N, Beral V, Burton P, Danesh J, et al. UK biobank: an open access resource for identifying the causes of a wide range of complex diseases of middle and old age. PLoS medicine. 2015 Mar;12(3):e1001779–e1001779.

12. Glodny B, Unterholzner V, Taferner B, Hofmann KJ, Rehder P, Strasak A, et al. Normal kidney size and its influencing factors - a 64-slice MDCT study of 1.040 asymptomatic patients. BMC Urol. 2009 Dec 23;9:19.

13. Bachtiar V, Kelly MD, Wilman HR, Jacobs J, Newbould R, Kelly CJ, et al. Repeatability and reproducibility of multiparametric magnetic resonance imaging of the liver. PLoS One [Internet]. 2019 Apr 10 [cited 2020 Jun 1];14(4). Available from: https://www.ncbi.nlm.nih.gov/pmc/articles/PMC6457552/
